# 4D printing through vat photopolymerization of two-stage UV-curable liquid crystal elastomers

**DOI:** 10.1038/s41467-026-68370-y

**Published:** 2026-01-15

**Authors:** Huan Jiang, Christopher Chung, Alston X. Gracego, James Breedlove, Yuchen Ding, Xiao Kuang, Martin L. Dunn, Kai Yu

**Affiliations:** 1 Department of Mechanical Engineering, University of Colorado Denver, Denver, CO USA; 2 Department of Mechanical Engineering, University of Wisconsin-Madison, Madison, WI USA

**Keywords:** Liquid crystals, Polymers, Design, synthesis and processing

## Abstract

Liquid crystal elastomers (LCEs) offer significant promise as soft actuator materials, but their potential has not yet been fully explored for 4D printing applications. Most existing studies rely on extrusion-based printing methods, which offer limited resolution and impose constraints on fabricating intricate, free-standing structures. Moreover, it remains a significant challenge to design and spatially control liquid crystal orientation within complex 3D structures to achieve desired shape transformations. To address these challenges, this study introduces a 4D printing strategy that combines two-stage UV-curable LCEs with vat photopolymerization-based 3D printing, such as digital light processing (DLP). The LCE can be initially printed into complex geometries with high precision, followed by a post-printing programming step in which mechanical deformation is applied to the printed structure to define the desired shape. A subsequent thermal treatment forms covalent linkages to lock the programmed configuration. The resulting structures can reversibly transition between the printed and programmed configurations upon temperature change. This 4D printing strategy is shown to overcome key limitations of current approaches and significantly broaden the design space and functional potential of programmable shape-changing structures for various applications, including mechanically active metamaterials, morphing architecture, and soft robotics.

## Abstract (web summary)

Liquid crystal elastomers have potential as 4D-printing actuators, but it is difficult to achieve complex shape changes in structures. Here, the authors report a 4D-printing strategy that combines vat polymerization-based 3D printing with a two-stage UV-curable resin.

## Introduction

4D printing of shape-changing polymers enables the creation of structures that actively respond to external stimuli, such as heat, light, humidity, and electric fields. The ability to program shape transformations during or after the printing process significantly expands the functionality of these materials across a broad range of applications, including morphing structures, soft robotics, biomedical devices, and aerospace components^1–4^.

A variety of active polymers have been investigated for 4D printing, including shape memory polymers (SMPs)^5–7^, hydrogels^8–10^, magnetic-responsive composites^11–13^, and liquid crystal elastomers (LCEs)^14–21^. Among these, LCEs are particularly attractive due to their rapid and reversible actuation behavior. When programmed into a monodomain state with aligned mesogens (typically composed of two or three linked benzene rings), LCEs can undergo reversible deformation in response to temperature changes. To date, most reported studies on 4D printing of LCEs have used extrusion-based methods, such as direct ink writing (DIW)^22–25^, where the viscous flow of resin through a nozzle generates shear forces that align mesogens along the filament’s axial direction.

However, DIW-based 4D printing faces persistent manufacturing constraints. The printing resolution is inherently constrained by the nozzle size (typically 0.5–2 mm)^26^, which restricts the ability to fabricate fine structural details. Because filaments must be deposited onto a substrate or existing layers, it remains difficult to print intricate free-standing or hollow geometries without support materials. Additionally, it is challenging to design and control mesogen alignment at the microscale within complex 3D architectures to achieve desired global shape changes. Consequently, most current 4D-printed LCE structures are limited to simple geometries and basic deformation modes, such as uniaxial contraction or folding. The full potential of LCEs as transformative actuating materials for 4D printing remains underexplored.

In contrast, vat photopolymerization-based 3D printing methods, such as stereolithography and digital light processing (DLP), cure resin layer by layer and offer high resolution for fabricating complex structures^19,27–29^. However, these methods do not inherently support in situ mesogen alignment during printing. While recent studies have explored the use of external fields, such as electric or magnetic fields^30,31^, to align mesogens within printed layers, these approaches require sophisticated hardware to generate and precisely control the fields. Due to their early-stage development, it remains unclear how effectively these techniques can program spatial mesogen alignment across printing layers in geometrically complex LCE structures.

An alternative strategy for programming shape change in LCEs is post-printing mechanical deformation, a concept originally adopted in early demonstrations of SMP-based 4D printing. Instead of aligning mesogens in situ during printing, users directly define global shape-change patterns by applying macroscopic deformation to the printed structure. Mesogen alignment at the microscale is then induced in response to the structural deformation. This strategy is particularly advantageous for fabricating complex 4D structures, where designing and controlling microscale mesogen alignment during printing are both challenging.

Despite its advantages, a key barrier to this 4D printing strategy is the lack of a suitable LCE system compatible with the post-printing mechanical programming. Most existing studies on 4D printing of LCEs rely on a thiol-acrylate system^15,32^, in which thermally triggered Michael addition reactions initially form oligomers or loosely crosslinked networks. Mechanical deformation (or the shear flow during DIW) is then used to program the monodomain state, followed by UV irradiation to cure the remaining acrylate groups and lock the mesogen alignment for reversible actuation. However, when such material is applied to the vat photopolymerization-based 3D printing, the initial UV polymerization rapidly consumes all reactive thiol and acrylate groups within seconds, leaving no functional groups available to fix the mesogen alignment after mechanical programming. Although recent studies have explored the use of non-LCE linkages to flexibly tune thermal-mechanical properties^33–36^, these linkages are still UV-curable and thus incompatible with post-printing programming strategies.

To address this limitation, this study introduces a two-stage UV-curable LCE system that incorporates both UV-curable acrylate and thermally curable epoxy groups. During the initial UV polymerization, the acrylate groups react to form a crosslinked network, while the epoxy groups remain unreacted. After printing, mechanical loading is applied to program the desired shape-change pattern, followed by thermal treatment to form epoxy linkages that lock the mesogen orientation. This approach essentially reverses the sequence of stimuli in conventional fabrication-programming steps of LCE networks (switching to UV first and then thermal), which makes it ideal for the proposed 4D printing strategy.

The study reveals how the stoichiometric ratio between the acrylate and epoxy groups enables widely tunable thermomechanical and actuation properties in the developed LCE system. The optimized resin formulation is then implemented in DLP-based 4D printing to fabricate complex LCE structures. The results demonstrate the material’s applicability across a broad range of 4D printing applications, including morphing structures, soft robotics, and active metamaterials. By addressing key limitations of the current LCE-based 4D printing methods, this work advances the state of the art and expands the design space for programmable shape-changing materials. Importantly, all precursor materials used in this LCE system are commercially available, making this strategy highly accessible to researchers interested in LCE-based 4D printing for diverse applications.

## Results and discussion

### DLP 4D printing and thermomechanical properties of printed LCEs

**Fig. 1:**
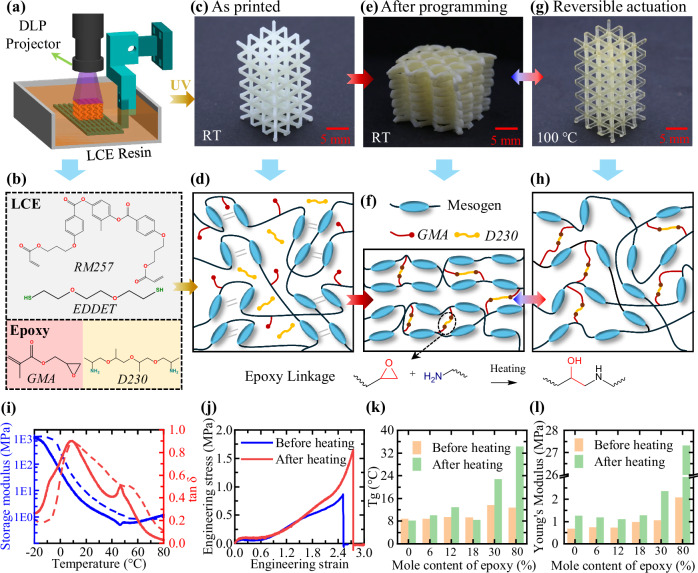
Printing setup, material composition, and thermomechanical properties. **a** Schematic of the digital light processing (DLP) printing setup. **b** Chemical structures of precursor resins for the two-stage liquid crystal elastomer (LCE), including the diacrylate monomer 1,4-bis[4-(3-acryloyloxypropyloxy) benzoyloxy]−2-methylbenzene (RM257), the dithiol spacer 2,2’-(ethylenedioxy) diethanethiol (EDDET), the monomer glycidyl methacrylate (GMA), and the diamine crosslinker poly (propylene glycol) bis (2-aminopropyl ether) (D230). **c** As-printed LCE lattice structure and **d** its corresponding network schematic. **e** After being heated at 80 °C for 14 h, the lattice retains its programmed shape at room temperature. **f** Schematic of the LCE network structure after post-heating, highlighting the formation of the epoxy linkages. **g** The programmed LCE lattice fully recovers the as-printed shape at 100 °C. **h** Schematic of the network structure after shape recovery. **i** Storage modulus and tanδ of AE_6 specimen. Solid curves: before heating. Dashed curves: after heating. **j** Room-temperature stress-strain curve for AE_6 specimen before and after thermal treatment. **k** Summary of glass transition temperature (T_g_) and **l** Young’s modulus of different LCE samples before and after thermal treatment. In **k**, **l**, each bar represents a single measurement (*n* = 1).

A customized DLP setup equipped with a 405 nm optical engine (Wintech, Carlsbad, CA) was used to fabricate LCE structures (Fig. 1a). Figure 1b shows the chemicals used to prepare the printable inks, including the diacrylate mesogen monomer 1,4-bis[4-(3-acryloyloxypropyloxy) benzoyloxy]−2-methylbenzene (RM257), the dithiol spacer 2,2’-(ethylenedioxy) diethanethiol (EDDET), the monomer glycidyl methacrylate (GMA), and the diamine cross-linker poly (propylene glycol) bis (2-aminopropyl ether) (D230). Diphenyl(2,4,6-trimethylbenzoyl)phosphine oxide (TPO) was used as the photo-initiator for UV polymerization. Detailed ink preparation and DLP printing procedures are provided in the “Methods” Section.

The adopted DLP printer not only enables the fabrication of intricate LCE components without the need for support materials but also offers a much higher printing resolution compared to the conventional DIW method. For example, as shown in the Supplementary Materials (Section S1), the smallest printable feature size is approximately 0.3 mm. As a demonstration, a polydomain LCE lattice structure was printed (Fig. 1c) using a resin formulation with a molar ratio of 1:0.06 between the acrylate and epoxy groups. The corresponding network structure is illustrated in Fig. 1d. At this stage, thiol-acrylate reactions formed a loosely crosslinked network upon UV irradiation, where GMA was covalently bonded to the network at one end, while D230 remained unreacted and isolated within the network.

After printing, the LCE structures were placed in a UV oven (20 mW/cm^2^) to ensure complete polymerization of the acrylate bonds. In the programming stage, the printed lattice structure was compressed to 50% global engineering strain, which drove the internal mesogens at each point of the printed LCE structures to align along the local principal stress direction^37^. Because different regions of the structure experience different principal stress states, the mesogen alignment directions varied spatially. Subsequently, the temperature was increased to form epoxy linkages. The programming temperature must be carefully selected, which should not only allow deformation of the material but also activate the epoxy–amine reaction so that the programmed shape is fixed. An increase in programming temperature accelerates the reaction rate according to the Arrhenius law and thus improves programming efficiency. However, excessively high temperatures may cause thermal degradation of the elastomeric LCEs. Thermogravimetric analysis (TGA) in the Supplementary Materials (Section S2) shows that degradation starts at approximately 330 °C. In this study, a relatively low processing temperature of 80 °C was selected to avoid the risk of thermal degradation while still providing sufficient reaction kinetics for effective programming.

It is noted that during the programming stage, the global deformation is fixed, which stabilizes the microscale network structure and chain configurations. Although heating above the phase transition temperature reduces the intermolecular interactions among the mesogens, their orientations and positions remain largely unchanged because the chain configurations and network structure are constrained by the imposed displacement boundary conditions. As a result, the overall spatial mesogen alignment is maintained during the thermal curing process. After 14 h of thermal treatment, the lattice retained its programmed deformation at room temperature after unloading (Fig. 1e). This complete shape fixity was attributed to the ring-opening reaction between GMA and the unreacted D230, which formed stress-free epoxy linkages (Fig. 1f). GMA was covalently bonded to both the acrylate network and epoxy linkages as a molecular bridge.

In the Supplementary Materials (Section S3), systematic Fourier-transform infrared spectroscopy (FTIR) analyses were conducted to substantiate the two-stage polymerization mechanism. Model reactions were carefully designed to examine the behavior of acrylate, thiol, and epoxy groups during resin storage, UV polymerization during DLP printing, and thermal treatment after printing. The results confirm that during resin storage, no unintended side reactions were detected for up to 21 h. The printable ink remains chemically stable, likely due to the presence of toluene that suppresses unintended side reactions by diluting the reactive species. Upon UV irradiation, reactions predominantly occur between the C=C bonds and thiol groups, which form the crosslinked LCE network, while the epoxy groups remain fully preserved. In the second-stage thermal polymerization, the epoxy–amine reaction becomes active, generating additional covalent linkages that stabilize the programmed deformation of the printed LCE structures.

When heated to 100 °C, the programmed LCE lattice fully recovered its original, as-printed shape (Fig. 1g) due to the nematic-isotropic phase transition. During this transition, the microscale mesogen alignment was disrupted, allowing the entropy elasticity of the acrylate network to overcome the elasticity of the epoxy linkages and drive the shape recovery (Fig. 1h). Upon cooling back to room temperature, the isotropic–nematic transition restored mesogen alignment and enabled the lattice to return to its the programmed shape. Although the printed LCE recovers its as-printed macroscopic configuration upon heating, the two states differ in their molecular structures: the high-temperature state corresponds to the isotropic phase, whereas the LCE immediately after printing is in the polydomain state.

As illustrated in the Supplementary Materials (Section S4), the programming and actuation procedures of the developed LCE system resemble those of conventional SMPs^38,39^. However, due to the distinct underlying mechanisms, this system exhibits fully reversible actuation behavior. Notably, the two configurations of the printed LCE structure during reversible actuation are explicitly defined by the user rather than being arbitrary: the high-temperature configuration corresponds to the CAD model used for 3D printing, while the room-temperature configuration is determined by the applied global programming deformation.

It is intriguing to investigate how the incorporation of epoxy linkages influences the thermomechanical behavior of LCEs. The thermal transitions and room-temperature stress-strain responses of a printed LCE specimen were characterized using dynamic mechanical analysis (DMA) and uniaxial tension testing, respectively. No programming deformation was applied during the heat treatments, so the LCE material remained in the polydomain state. As shown in Fig. 1i, j, the addition of 6 mol% epoxy moieties (relative to acrylate content) increases the storage modulus and significantly improves the failure strength from 0.75 to 1.7 MPa. Despite this enhancement, the material remains soft and highly stretchable at room temperature. The glass transition temperature (T_g_), identified by the first peak on the tanδ curve, remains unchanged at approximately 8 °C. Meanwhile, the phase transition temperature (T_i_), indicated by the second peak, increases only slightly from 45 to 50 °C. This characteristic is advantageous for applications such as soft robotics, as the introduction of the epoxy linkages enhances stiffness, strength, and toughness without significantly altering the thermodynamics of actuation.

As detailed in the Supplementary Materials (Section S5 and Table S1), a series of LCE specimens were printed with varying epoxy content, ranging from 1 mol% to 80 mol%. These samples are referred to throughout this study as AE_X, where *X* denotes the molar percentage of epoxy moieties relative to the acrylate network. The AE_0 specimen serves as the control, representing a thiol-acrylate LCE without epoxy linkages. Across all LCE samples, there is an excess of acrylate relative to the thiol groups. As shown in the FTIR characterizations in the Supplementary Materials (Section S3), during UV polymerization, the acrylate bonds exhibit higher reactivity than the thiol groups. An excess amount of acrylate helps minimize residual thiol groups, which are not required for the second-stage thermal polymerization.

The T_g_ and room-temperature moduli of these samples are summarized in Fig. 1k, l, respectively. For AE_0, AE_6, and AE_12, their T_g_ before thermal treatment remains consistent at approximately 8–10 °C (also see Supplementary Materials, Section S6), and the moduli range from approximately 0.5 to 1 MPa. Post-heating results in only slight increases in these values, indicating that the LCEs retain their compliance as soft elastomers at room temperature. In contrast, with higher epoxy content, AE_30 and AE_80 exhibit pronounced stiffening after thermal treatment, with T_g_ exceeding room temperature. This mechanical enhancement suggests their potential for use as load-bearing actuating materials that maintain structural stiffness under ambient conditions.

### Reversible actuation behaviors of printed LCEs

**Fig. 2:**
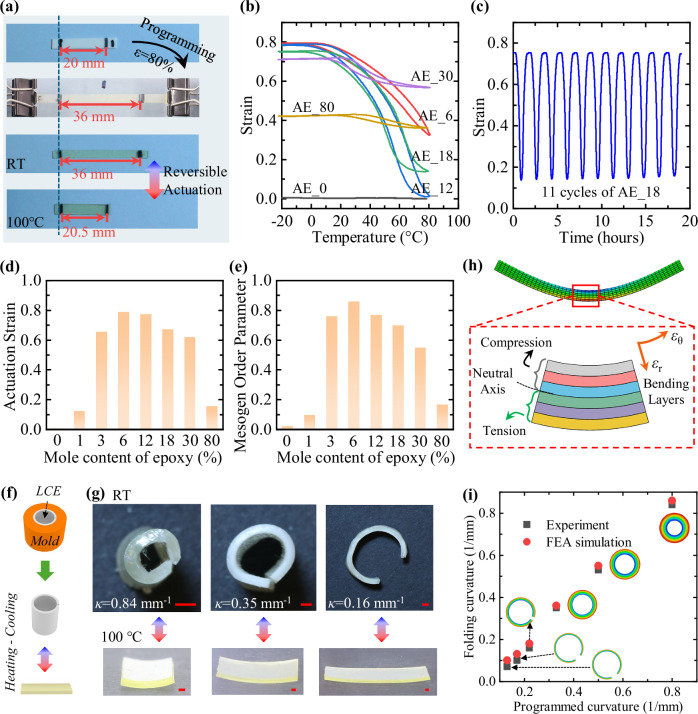
Reversible actuation behaviors of LCEs. **a** Evolution of sample length during programming and reversible actuation stages between room temperature (RT) and 100 °C. The programming strain ε = 80%. **b** Reversible actuation strain of samples with different epoxy contents tested within a fixed temperature range. **c** Repeatability of actuation strain in sample AE_18 with consistent performance over 11 cycles. **d** Actuation strain amplitudes of liquid crystal elastomer (LCE) samples with different epoxy content. **e** Room-temperature mesogen order parameters of samples after programming. **f** Schematic view showing the programming of folding LCE specimen. **g** Reversible folding deformation of AE_6 under different programming folding curvatures, κ. Scale bar = 1 cm. **h** Schematic illustration of the modeling approach for reversible folding deformation in LCE strips. ε_θ_ and ε_r_ denote the axial strain and the thickness strain of the bending layers, respectively. **i** Comparison of folding curvature between experiment and finite element analysis (FEA) simulation. In **d**, **e**, each bar represents a single measurement (n = 1).

The actuation behavior of the LCE specimen AE_6 was first characterized by tracking the distance between two marking points during the programming and actuation stages (Fig. 2a). After printing, the initial separation of these two points was L_0_ = 20 mm. The sample was then stretched to 80% programming strain and fixed in this deformed state during thermal treatment at 80 °C for 14 h. After unloading at room temperature, the distance between the two marking points remained at 36 mm (identical to the programmed length), which indicated a 100% shape fixity. Upon heating to 100 °C, the distance decreased to L = 20.5 mm, which corresponded to a shape recovery ratio of 97.5%, calculated as 1−(L−L_0_)/L_0_.

The shape fixity of other LCE specimens with varying epoxy content was measured using the same method. It is found that AE_0 exhibited no shape fixity, while AE_1 showed low fixity values below 60% due to insufficient epoxy linkages to lock the deformed acrylate network and aligned mesogens. In contrast, AE_3 through AE_30 demonstrated excellent shape fixities exceeding 95%. However, the shape fixity of AE_80 decreased to 84%, likely due to the high epoxy content diluting the mesogen concentration. This reduction weakened the intermolecular attractions among mesogens at room temperature and partially released the entropy elasticity stored in the acrylate network, leading to spring-back deformation after unloading.

After programming, the evolution of actuation strain in AE_6–AE_80 samples was examined using DMA. Tests were conducted over a temperature range of –20 to 80 °C at a heating rate of 1 °C min^−1^, as shown in Fig. 2b. Upon heating, the onset temperature of shape recovery varied with epoxy content. AE_6–AE_18 samples exhibited nearly identical onset temperatures around 15 °C, while AE_30 and AE_80 showed higher onset temperatures of approximately 30 and 40 °C, respectively. This indicates that epoxy contents above 30% have a significant influence on the thermomechanical properties of LCEs.

During cooling, all specimens fully recovered their initial lengths, and this reversible shape-change behavior was highly repeatable over multiple heating–cooling cycles. For instance, AE_18 was subjected to 11 consecutive cycles and exhibited consistent strain evolution without noticeable degradation (Fig. 2c).

Figure 2d summarizes the actuation strain amplitudes, which were obtained by heating the LCE samples at 80 °C for over 30 min until the strain reached saturation (Supplementary Materials, Section S7). The results show that the AE_1 specimen exhibited a much lower actuation strain due to its limited shape fixity, whereas AE_3–AE_18 samples achieved actuation strains close to the target programming strain. Increasing the epoxy content beyond 30% led to a pronounced reduction in actuation strain; for example, AE_80 showed a strain amplitude of only ~18%. This sample is not able to fully recover its initial printed geometry because the newly formed epoxy linkages during programming are stress-free; at high concentrations, these linkages strongly resist the entropy-driven recovery of the deformed acrylate network.

The variation in actuation strain amplitude can be attributed to the different degrees of mesogen alignment induced during the programming step. As described in the Supplementary Materials (Section S8), polarized FTIR measurements were performed to quantify alignment^37,40–42^. The bending mode of C─H bonds on the benzene ring exhibits the highest absorption peak when the light polarization is parallel to the mesogen alignment and the lowest when perpendicular. The difference between these extreme FTIR peaks defines the mesogen alignment degree, ranging from 0 to 1. As summarized in Fig. 2e, the negligible order parameter of the control AE_0 sample confirms randomly aligned mesogens in polydomain state. With increasing epoxy content, the order parameters of the other samples initially increase and then decrease, following the same trend as the actuation strain amplitudes.

In addition to axial actuation, folding deformation is a widely employed mechanism in 4D-printed structures. To characterize this behavior, AE_6 LCE samples with a thickness of 0.7 mm were placed inside cylindrical molds of varying radii and thermally treated at 80 °C for 14 h (Fig. 2f). Figure 2g shows the programmed shapes of several samples observed at room temperature. Additional examples are provided in the Supplementary Materials (Section S9). The ability of LCEs to retain their programmed folding curvature depends on the ratio of sample thickness to folding radius: a higher ratio induces greater axial strain within the bending layers, which promotes monodomain mesogen alignment and improves shape fixation. Experimental results indicate that shape fixity exceeds 90% when the programmed folding curvature is greater than 0.18 mm^−1^. Notably, this is a moderate threshold, as the corresponding folding radius (5.5 mm) remains much larger than the sample thickness (0.7 mm). In practical 4D-printed designs, folding curvatures are often much higher, with radii comparable to the structural feature size. Therefore, the LCE material is expected to maintain excellent shape fixity across most shape-changing structural applications. Regardless of the programmed folding curvature, all samples fully recovered their original flat geometry upon reheating.

The reversible folding curvature can be quantitatively linked to the uniaxial actuation strain of the same LCE material. As detailed in the Supplementary Materials (Section  S9), a finite element analysis (FEA) model was developed to simulate folding actuation. In this model, the LCE sample was discretized into multiple bending layers (Fig. 2h), each subjected to a different axial programming strain determined by the imposed folding curvature. This axial programming strain determines the corresponding actuation strain using an experimentally derived relationship. The resulting actuation strain of each layer was then used to assign a unique anisotropic thermal expansion coefficient to each bending layer, which collectively governs the overall folding behavior of the sample upon temperature change. As shown in Fig. 2i, the FEA predictions closely match the experimental results, which confirm that folding deformation can be fundamentally interpreted as an extension of axial actuation behavior distributed across the bending layers.

### 4D printed active mechanical metamaterials

Based on the characterizations presented in the previous sections, the AE_6 specimen was strategically selected for the following studies, as it exhibited nearly 100% shape fixity and recovery ratio. The incorporated epoxy linkages enhanced the material’s toughness without significantly affecting the thermodynamics of the phase transition.

Mechanical metamaterials are architected structures composed of repeating unit cells engineered to exhibit tunable mechanical behavior. Prior research has largely focused on their passive properties, such as stiffness modulation through geometric design and energy dissipation via buckling, structural instabilities, or negative Poisson’s ratio mechanisms. By integrating LCE-based 4D printing, these metamaterials acquire an additional functional dimension, i.e., active responsiveness to temperature changes, which opens opportunities for adaptive systems that were previously unattainable yet highly desirable.

**Fig. 3:**
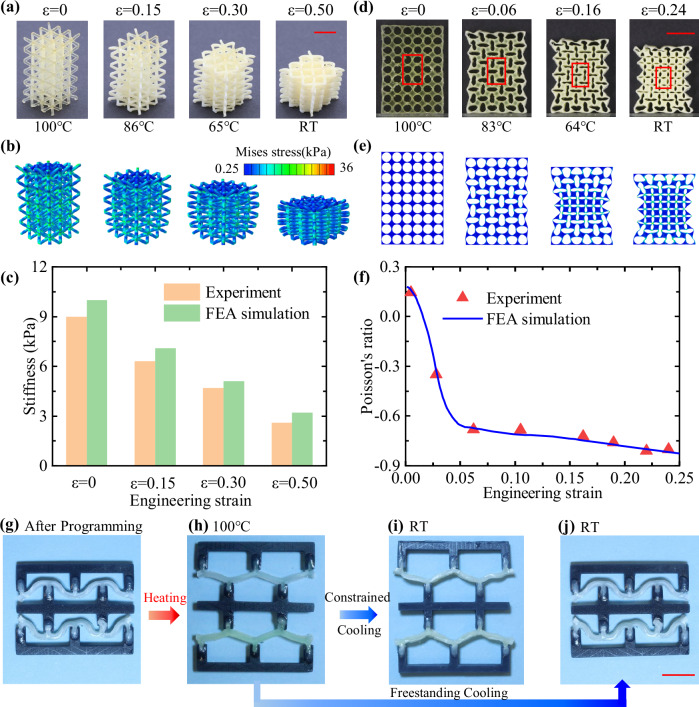
4D printed active mechanical metamaterials. **a** Shape changing of the lattice at different temperatures. Scale bar = 1 cm. ε denotes the engineering strain of the structure. **b** Finite element analysis (FEA) simulations to determine the stiffness of the lattice at strain levels equivalent to the actuation strain. **c** Lattice stiffness variation due to reversible actuation and mechanical deformation. Each bar represents a single measurement (n = 1). **d** Reversible shape changing of the auxetic metamaterial at different temperatures. The highlighted area is used to characterize the negative Poisson’s ratio. Scale bar = 1 cm. **e** FEA simulations to determine the Poisson’s ratio of auxetic metamaterials at strain levels equivalent to the actuation strain. **f** The comparison of Poisson’s ratio between experiment and FEA simulations. **g** Programmed configuration of bistable metamaterial. **h** Recovered shape of bistable metamaterial upon heating. **i** The configuration of bistable metamaterial at room temperature (RT) with displacement constraint. **j** The configuration of bistable metamaterial at RT with stress-free condition. Scale bar = 1 cm.

As shown in Fig. 3a, a truss lattice was printed using AE_6 LCE and programmed under 50% compressive strain. The lattice exhibited nearly 100% shape fixity after unloading at room temperature and fully recovered its original printed configuration upon heating to 100 °C. Notably, the actuation strain remained stable at each temperature, which provided exciting opportunities to actively modulate the lattice geometry and stiffness simply by adjusting the temperature, eliminating the need to print new lattices for different stiffness requirements. For example, small-strain uniaxial compression tests at each temperature demonstrated that the lattice stiffness could be tuned threefold, from approximately 3 kPa at room temperature to around 9 kPa at 100 °C.

FEA simulations were conducted to verify that the stiffness of the LCE lattice at different temperatures is equivalent to that of stress-free lattices printed in the same geometry. As described in Supplementary Materials (Section S10), the FEA models were compressed to specific strain levels, and the resulting node coordinates were extracted. These coordinates were then used to generate new FEA models in identical geometries but without internal stress distributions. The stiffness of these stress-free lattices was then evaluated (Fig. 3b). As shown in Fig. 3c, the simulated stiffness closely matches that of the thermally actuated lattice under the same global strain. This result indicates that the LCE lattices, regardless of temperature or actuation history, behave like newly fabricated structures without residual stress. Such a capability allows a single lattice design to fulfill different stiffness requirements without the need for re-fabrication for each application scenario.

Auxetic metamaterials with a negative Poisson’s ratio have been extensively studied for their ability to enhance shear resistance, indentation resistance, fracture toughness, and energy absorption. Building on the concept of elastic instability^43^, an auxetic structure was printed using AE_6 LCE, as shown in Fig. 3d. Then, the structure was programmed with 24% compressive strain. During the compression programming of the auxetic metamaterials, no noticeable structural instability was observed in this study. However, for structures whose transverse dimension is much smaller than their height, global buckling may occur. To address this issue, one possible approach is to apply transverse stretching instead of compression to achieve an equivalent global programming deformation. Alternatively, a standardized method is to use an anti-buckling compression fixture (e.g., ASTM D695), which employs a rigid frame to provide continuous lateral support and ensure uniform transverse displacement while preventing localized bulging or buckling.

After programming, the structure exhibited reversible and repeatable shape changes during heating–cooling cycles (Supplementary Movie 1). Similarly, this capability opens opportunities for tuning porosity, Poisson’s ratio, and overall geometry of printed structures through temperature control, which in turn allows real-time adjustment of their stiffness, toughness, and energy absorption capacity.

Beyond their mechanical properties, auxetic metamaterials have also been explored for a range of functional applications, such as tunable acoustic bandgaps^44^, tailorable thermal expansion behavior^45^, adaptive fillers^46^, and enhanced electromechanical coupling^47^. Traditionally, achieving these functionalities requires external mechanical loading to actively control the Poisson’s ratio and structural geometry. Here, with the reversible shape-changing capability of the LCE material, this dependence on external force is no longer needed. Instead, temperature-driven actuation provides an equivalent means of controlling these physical properties. For instance, Fig. 3e presents FEA simulations of an elastic auxetic metamaterial with mechanical compression. The results show that when the applied strain equals the thermal actuation strain, the structure exhibits the same geometry and Poisson’s ratio, as shown in Fig. 3f. Since temperature control and mechanical control are functionally equivalent, existing design principles, optimizations, and theoretical models developed for auxetic metamaterials can be directly applied to the LCE-based structures.

Finally, we demonstrate that two-stage LCEs can expand the functional capabilities of bistable metamaterials, which have been widely studied for energy absorption and energy trapping applications^48,49^. As shown in Supplementary Materials (Section S11), two sine-curved LCE beams were 3D printed using AE_6 and assembled with 3D-printed frames to form a bistable metamaterial unit cell. The cell was then compressed by 5 mm and held in this deformed state during thermal treatment to program the LCE beams. Figure 3g shows the fixed configuration after unloading at room temperature.

Upon heating, the programmed structure was able to overcome the energy barrier and recovered its original, as-printed shape (Fig. 3h). During subsequent cooling, if the frame displacement was constrained, the structure remained in the recovered configuration even after the boundary conditions were removed at room temperature, as shown in Fig. 3i. This behavior offers a practical solution for repeated energy absorption in applications such as impact protection and energy-absorbing systems (e.g., automotive bumpers). After undergoing buckling deformation, the structure can be reset simply by heating, followed by cooling under appropriate constraints to preserve the desired configuration for the next mechanical loading cycle. This characteristic is beneficial for addressing a persistent challenge in the field, where conventional bistable structures often require external force to return to their initial state after buckling.

In contrast, if no boundary constraints are applied during the cooling step, the structure freely returns to its programmed configuration (Fig. 3j), albeit at a slower rate. The structure can repeatedly switch between its two stable configurations in response to temperature changes. As shown in Supplementary Movie 2, the shape transition during heating occurs rapidly, indicating that a significant amount of stored mechanical energy is released within a short period. This behavior enables potential applications in cyclic energy trapping and release, such as bio-inspired jumping robots^50^, where thermal energy can be cyclically converted into mechanical motion.

It is important to note that whether the structure can overcome the energy barrier during heating depends on the programming displacement. As shown in Supplementary Materials (Section S11) and Supplementary Movie 3, when the programming displacement was increased to 7 mm, the structure was unable to recover its printed shape. This observation highlights an intriguing mechanics problem that warrants further investigation.

### 4D printed reversible morphing structures and soft robots

**Fig. 4:**
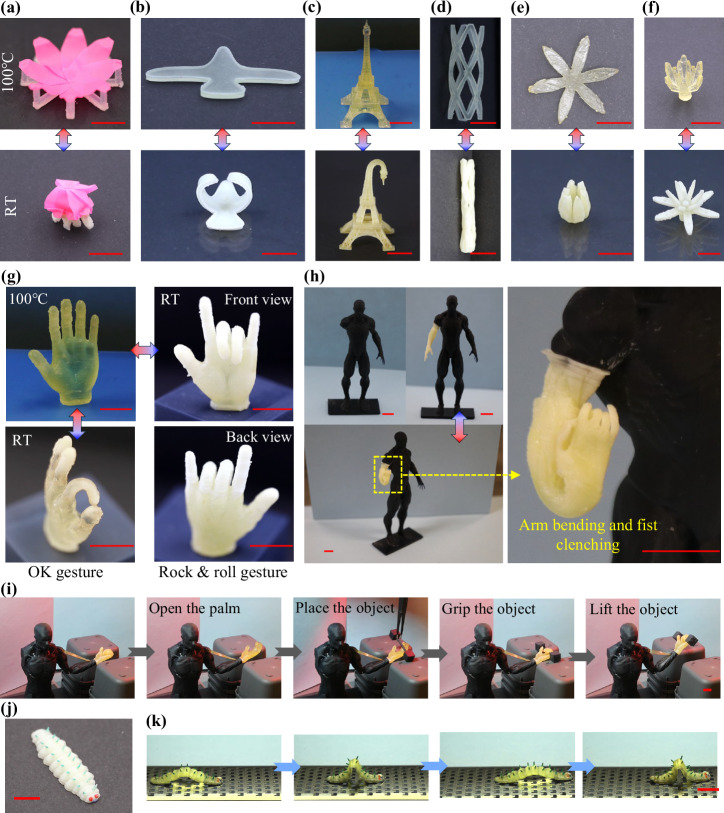
Demonstration of reversible shape-changing structures and soft robotic systems when the temperature changes between room temperature (RT) and 100 °C. **a** A deployable antenna model, **b** morphing aircraft wings, **c** a 4D-printed Eiffel Tower model, **d** a medical stent, **e** a single-layer flower, and **f** a double-layer flower in blossom. **g** 4D-printed hand models showing different gestures in response to temperature changes. **h** A prosthetic arm demonstrating reversible actuation, with elbow bending and hand contraction into a fist. **i** A simplified soft robotic system that grips and lifts a dumbbell. **j** An inchworm soft robot in the as-printed configuration. **k** Inchworm-like crawling motion under successive heating and cooling cycles. Scale bars in all panels are 1 cm.

4D printing has enabled the creation of morphing structures with promising applications in astronautics, aerospace, biomedical, and consumer products. The developed two-stage LCE facilitates the fabrication of complex 3D structures with precisely programmable and fully reversible shape-changing behavior. In this section, we demonstrate several representative 4D-printed structures using AE_6 LCE (Fig. 4a–f). Specifically, Fig. 4a shows a deployable antenna, where a pink reflector film is supported by an LCE-based base structure. Figure 4b presents a morphing aircraft model, where the wings fold upon cooling to mimic the flight of birds. Figure 4c shows a 4D-printed Eiffel Tower with intricate details and a minimum feature size of ~0.25 mm. Figure 4d displays a printed vascular stent programmed to a smaller diameter for minimally invasive surgery. Finally, Fig. 4e, f illustrate single-layer and double-layer 4D-printed flowers, which bloom or contract upon heating. In each demonstration, the high-temperature configuration corresponds to the as-printed shape, while the low-temperature configuration represents the programmed state. The reversible shape changes of these structures during heating and cooling cycles are shown in Supplementary Movies 4–9.

While similar morphing structures have been reported in prior 4D printing studies, most have been based on one-way SMPs, which require external intervention to reset their shape after actuation. This study demonstrates comparable morphing structures that undergo fully reversible shape transitions using a two-stage LCE-based system. This capability is particularly advantageous for applications in environments where manual resetting is impractical or impossible, such as outer space, deep-sea exploration, and biomedical implants.

In addition to morphing structures, the developed two-stage LCE system enables advanced applications in reversible soft robotics. As shown in Fig. 4g, 3D-printed hand models were programmed to perform specific gestures, such as OK and rock and roll, by controlling the folding of individual fingers. These gestures are fully reversible through temperature cycling (Supplementary Movies 10 and 11). Figure 4h further extends this concept to a 3D-printed prosthetic arm composed of an upper arm, forearm, and hand. The arm was programmed to exhibit reversible actuation, such that the elbow bends and the hand contracts into a fist (Supplementary Movie 12), serving as a model of assistive devices for individuals with disabilities.

This combination of programmable muscle movement and finger-level control allows for functional soft robotic systems capable of performing simple tasks. As a proof-of-concept, Fig. 4i illustrates a simplified model representing a person with impaired arm and hand mobility. A printed LCE strip is attached to the forearm to act as an artificial muscle, while the hand is programmed to open and close reversibly. During operation, local heating is first applied to open the hand. A dumbbell is placed into the palm, and subsequent cooling enables the hand to grip the object firmly. Heating the LCE strip then lifts the forearm through a pin connection at the elbow. This actuation process is fully reversible: reheating the hand releases the object at a designated location. This motion sequence is demonstrated in Supplementary Movie 13.

Finally, the two-stage LCE system enables the crawling motion of soft robots. As shown in Fig. 4j, an inchworm-like soft robot was 4D-printed with intricate geometry and fine surface features, which are highlighted in different colors. The structure was then programmed with a folding deformation along its body (Supplementary Materials, Section S12) and placed on a substrate with one-way friction. Upon successive heating and cooling, the inchworm-like robot repeatedly folds and extends, which mimics biological crawling motion and propels itself forward. The time-lapsed crawling images are shown in Fig. 4k, and the crawling process is presented in Supplementary Movie 14.

In summary, this study introduces a two-stage UV-curable LCE system that addresses key challenges in the current field of 4D printing. Unlike existing LCE systems that are limited to extrusion-based printing, the material developed here undergoes initial UV polymerization followed by thermal treatment to form epoxy linkages that fix the programmed deformation. As a result, it is compatible with vat photopolymerization-based 4D printing and enables the fabrication of reversible, intricate LCE structures with minimal manufacturing constraints. Additionally, the material system allows users to directly program desired shape transformations through macroscopic deformation, eliminating the need for complex design and spatial control of mesogen orientation at the microscale.

This study demonstrates that the thermomechanical and actuation properties of the material are broadly tunable by varying the stoichiometric ratio between acrylate and epoxy groups. It further reveals the significantly enhanced and potentially transformative capabilities of printed LCE structures, including applications in mechanical metamaterials, morphing architectures, and soft robotics. In contrast to conventional 4D printing strategies, the structures produced in this work either feature intricate geometries that cannot be fabricated using DIW or exhibit reversible actuation behaviors that are not achievable with one-way SMPs. Moreover, all precursor materials used in the resin formulation are commercially available, making this strategy highly accessible for researchers seeking to develop advanced, reversible shape-changing systems across a wide range of applications.

## Methods

Ink preparation: the printable ink for LCE 4D printing was prepared using the diacrylate mesogen monomer 1,4-bis[4-(3-acryloyloxypropyloxy) benzoyloxy]−2-methylbenzene (RM257), the dithiol spacer monomer 2,2’-(ethylenedioxy) diethanethiol (EDDET), the monomer glycidyl methacrylate (GMA), and the diamine crosslinker poly (propylene glycol) bis (2-aminopropyl ether) (D230). Diphenyl(2,4,6-trimethylbenzoyl)phosphine oxide (TPO) was used as the photo initiator, and toluene was added as a non-reactive solvent to reduce the viscosity of the resin mixture for DLP printing. RM257 was purchased from Daken Chemical Limited (China), while the remaining chemicals were obtained from Sigma-Aldrich (St. Louis, MO, USA). All chemicals were used without further purification.

Among the four main components, RM257 and EDDET form the acrylate network upon UV irradiation, while GMA and D230 form the epoxy linkage upon thermal curing. For all ink formulations, the molar ratio between RM257 and EDDET was fixed at 1:0.87, and the molar ratio between GMA and D230 was fixed at 1:0.18. However, the overall molar ratio between the acrylate and epoxy groups varied across samples, ranging from 1:0 to 1:0.8. The detailed compositions of each ink formulation are provided in the Supplementary Materials (Table S1).

To prepare the printable ink, RM257, EDDET, TPO, and toluene were first mixed at their designated ratios in a glass vial and heated at 80 °C for 30 min to fully dissolve the solids. After removal from the oven, the mixture was allowed to cool to room temperature. GMA and D230 were then added in their respective ratios and thoroughly mixed. Any entrapped bubbles were removed by vacuum degassing. The final resin mixture was stored in a dark environment at room temperature for 12 h prior to being transferred into the DLP printer tank for printing.

DLP 4D printing: a customized bottom-up DLP setup equipped with a 405 nm optical engine (Wintech, Carlsbad, CA) was used for LCE 4D printing. A fluorinated ethylene propylene film was attached to the bottom of the resin vat to facilitate easy separation of cured layers. All CAD models used in this study were created in SolidWorks (Dassault Systemes, Waltham, MA), and the resulting STL files were sliced into 2D image layers using PrusaSlicer software (Prague, Czech Republic) with a layer thickness of 50 μm. During printing, the motion of the build platform was controlled by a motorized translation stage (MTS50-Z8, Thorlabs Ltd., Newton, NJ) connected to a DC motor controller (TDC001, Thorlabs Ltd., Newton, NJ). The printed layer thickness was maintained at 50 μm. The curing time for the initial layer was set to 30 s, while each subsequent layer was exposed for 10 s.

DMA characterizations: the thermomechanical properties of the printed LCE samples were characterized using a DMA tester (Model Q800, TA Instruments, New Castle, DE, USA). To assess the thermal transition behavior, the analysis was conducted at a frequency of 1 Hz with a strain amplitude of 1%. The temperature was first equilibrated at −20 °C for 10 min, followed by a continuous heating ramp at 1 °C/min up to 80 °C. The glass transition temperature was determined as the temperature corresponding to the first peak in the tanδ curve, while the phase transition temperature was identified as the second peak. The reversible shape deformation behavior was evaluated by heating free-standing LCE samples from −20 to 80 °C at 1 °C/min.

Mechanical characterizations: the mechanical properties of printed LCE specimens and metamaterial structures were characterized using a universal testing machine (MTS Criterion Model 41, MTS Systems Corp., Eden Prairie, MN, USA) equipped with a 500 N load cell and a temperature-controlled chamber. All tests were conducted under uniaxial loading conditions at designated temperatures. A constant loading rate of 6 mm/min was used to minimize rate-dependent viscoelastic effects.

Polarized FTIR: polarized FTIR was used to determine the mesogen order parameter of the printed LCE specimens. Measurements were conducted in transmission mode using a Nicolet iS50 FTIR spectrometer (Thermo Fisher Scientific, Waltham, MA, USA), with sample thickness kept below 0.5 mm. The instrument is equipped with a built-in ZnSe polarizer capable of rotating in 1° increments up to 180°.

During testing, the LCE sample remained fixed while the polarization angle of the incident light was rotated from 0° to 180° in 10° increments. At each orientation, FTIR spectra were collected in less than 2 s. The C–H bending vibrations on the benzene rings exhibited polarization-dependent absorption intensities. By analyzing the variation in these absorption peaks across different angles, the mesogen order parameters of the printed LCE specimens were calculated. Detailed data analysis is provided in the Supplementary Materials (Section S3).

## Supplementary information

Supplementary Information

Description of Additional Supplementary Files

Supplementary Movie 1

Supplementary Movie 2

Supplementary Movie 3

Supplementary Movie 4

Supplementary Movie 5

Supplementary Movie 6

Supplementary Movie 7

Supplementary Movie 8

Supplementary Movie 9

Supplementary Movie 10

Supplementary Movie 11

Supplementary Movie 12

Supplementary Movie 13

Supplementary Movie 14

## Source data

Source Data

Transparent Peer Review File

## Data Availability

All data generated in this study are provided in the Supplementary Information Source data file. Data are available from the corresponding author on request. Source data are provided with this paper.
