# Supplementary material for: 4D printing through vat photopolymerization of two-stage UV-curable liquid crystal elastomers
Source: Nat Commun. 2026 Jan 15;17:1671. doi: 10.1038/s41467-026-68370-y (PMC12910083; doi:10.1038/s41467-026-68370-y)
Supplement: Supplementary file 1 — Supplementary Information [file 41467_2026_68370_MOESM1_ESM.pdf]

Supplementary Information for

4D Printing through Vat Photopolymerization of Two-Stage UV-Curable Liquid Crystal Elastomers

Huan Jiang [1], Christopher Chung [1], Alston X. Gracego [1], James Breedlove [1], Yuchen Ding [1], Xiao Kuang [2], Martin L. Dunn [1, *], Kai Yu [1, *]

[1.] Department of Mechanical Engineering, University of Colorado Denver, Denver, CO 80217, USA

[2.] Department of Mechanical Engineering, University of Wisconsin-Madison, Madison, WI, 53706, USA

*Corresponding authors: KY: kai.2.yu@ucdenver.edu, MD: martin.dunn@ucdenver.edu

S1. Characterization of DLP Printing Resolution

To characterize the digital light processing (DLP) printing resolution, several cylinders with diameters ranging from 1.4 mm to 0.05 mm were printed. The corresponding CAD models and printed results are shown Figure S1. It is observed that the smallest feature that can be accurately fabricated is approximately 0.3 mm in diameter; cylinders below this size either cannot be printed or exhibited poor surface quality. This critical feature size is notably smaller than that achievable by direct ink writing (DIW) printing, where the printable resolution is primarily limited by the nozzle diameter, typically on the millimeter scale. It should also be noted that the printing resolution of DLP systems depends strongly on the printer hardware, particularly the light engine. In this work, a commercially available 405 nm optical engine (Wintech, Carlsbad, CA) is used, which provides a representative resolution for standard DLP setups. Higher resolutions could be achieved by employing optical engines with finer projection pixel sizes.

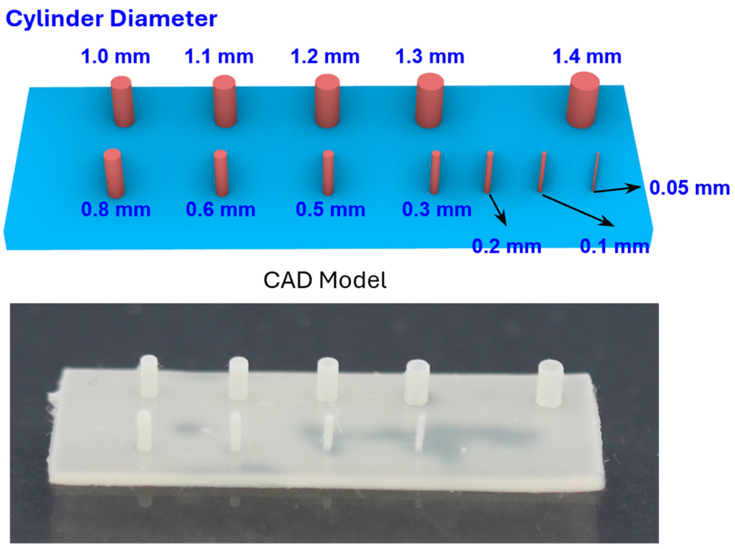

Figure S1. CAD models (top) and corresponding printed results (bottom) of cylindrical structures with diameters ranging from 1.4 mm to 0.05 mm.

S2. Thermogravimetric Analysis (TGA)

Thermogravimetric analysis (TGA) was performed on the LCE sample AE_6, with the temperature increased from room temperature to 600 °C at a rate of 10 °C min^{-1}. The change in normalized mass as a function of temperature is shown in Figure S2. The thermal degradation temperature, defined as the temperature corresponding to a 5% mass loss, is approximately 330 °C, which is significantly higher than the programming temperature used in this study.

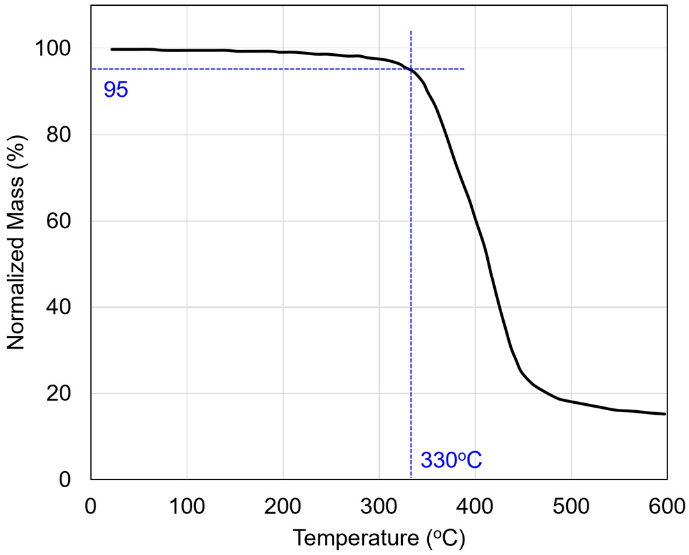

Figure S2. Normalized mass as a function of temperature during the TGA test.

S3. Fourier Transform Infrared Spectroscopy Characterizations

Fourier Transform Infrared Spectroscopy (FTIR) was conducted to investigate the reaction mechanisms involved in the two-stage polymerization of LCEs. To minimize peak overlap, spectral interference, and potential misinterpretation, most FTIR studies in this section were performed using a series of model reactions in which only selected chemical components were mixed, rather than analyzing the reactions of the complete resin system. All FTIR measurements were carried out on a Nicolet iS50 spectrometer (Thermo Fisher Scientific, Waltham, MA, USA) operated in attenuated total reflection (ATR) mode. In this mode, infrared light enters the liquid or solid sample through a high-refractive-index crystal and undergoes total internal reflection. The resulting evanescent wave penetrates the sample surface, and the reflected light exhibits characteristic absorption peaks corresponding to specific chemical bonds.

The FTIR characterizations were designed to examine potential chemical reactions occurring during the three major stages of the LCE printing process: (1) resin storage, (2) UV-induced polymerization during printing, and (3) thermal treatment of the printed LCE samples.

S3.1. Reactions during Resin Storage

We first examined whether any unintended side reactions occurred during the storage of the LCE ink under ambient conditions at room temperature or during printing when the resin was not exposed to UV light. Possible reactions include those between the epoxy rings of GMA and the amine groups of D230; reactions between epoxy rings and the thiol (–SH) groups of EDDET catalyzed by amines; and acrylate–thiol Michael addition reactions that may proceed in the presence of basic amines.

First, GMA and D230 were mixed at the same molar ratio as that used in the printable ink of the LCE samples (Figure S3a), and the mixture was stored in a transparent glass bottle at room temperature for different durations. Figure S3b shows the FTIR spectra in the range of 750–3500 cm^{-1}, and Figure S3c presents the vertically offset spectra for clearer comparison. Figure S3d highlights the characteristic epoxy ring absorption peak at 907 cm^{-1}. No noticeable changes were observed in any peaks, particularly in the epoxy peak, which indicates that no detectable reaction occurred between the epoxy and amine groups during the 21-hour storage period.

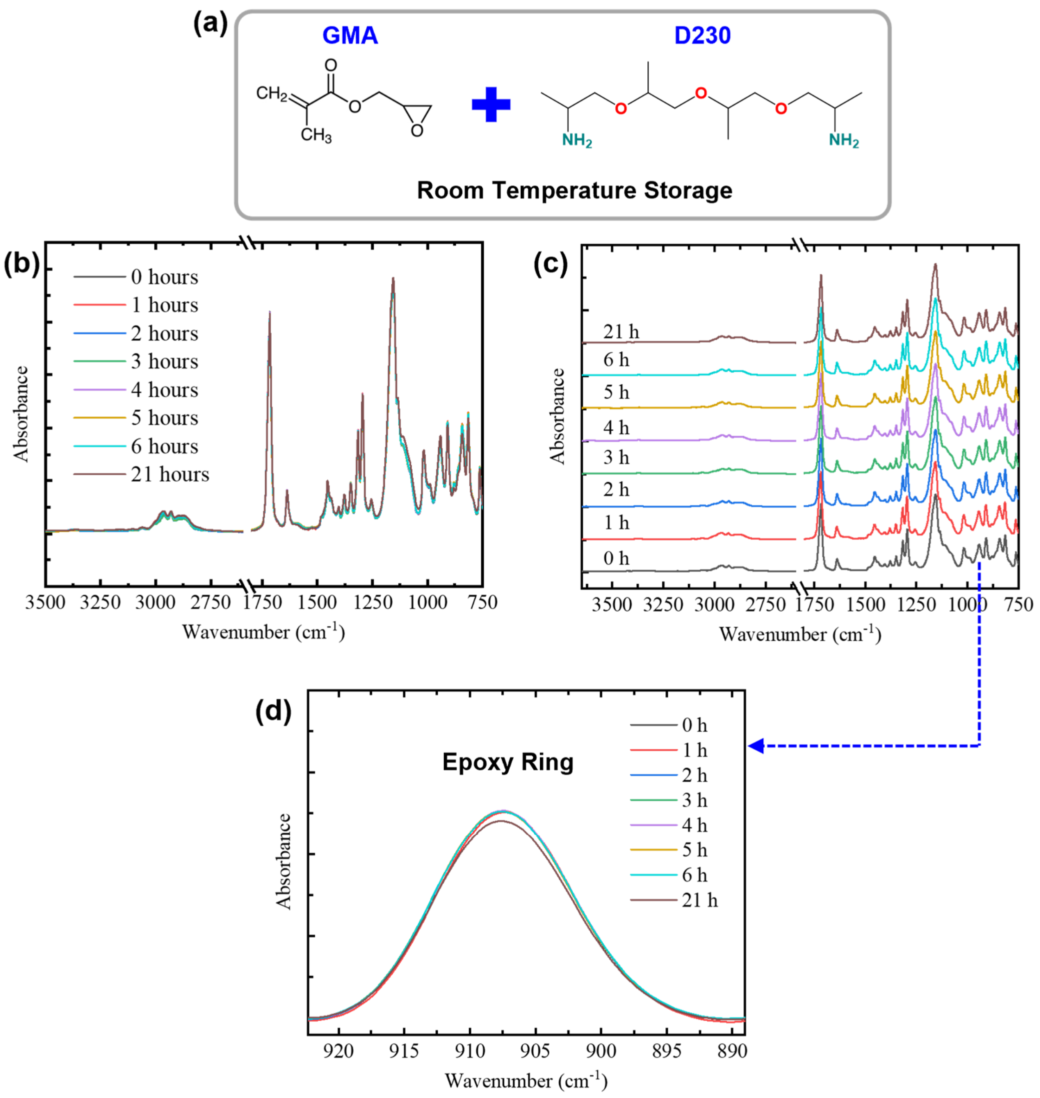

Figure S3. FTIR analysis of the model reaction between GMA and D230 at room temperature. (a) Chemical structures of the reactants. (b) FTIR spectra in the range of 750–3500 cm^{-1}. (c) Vertically offset spectra for clearer comparison. (d) Characteristic epoxy ring absorption peak at 907 cm^{-1}.

Next, GMA, D230, and EDDET were mixed at the same molar ratio as that used in the printable ink of the AE_80 sample, which contained the highest epoxy content (Figure S4a). In this system, the epoxy rings could potentially react with the thiol groups, with the amines serving as catalysts. The corresponding FTIR spectra (Figure S4b) and the vertically offset curves (Figure S4c) again show minimal changes. Figures S4d and S4e respectively highlight the absorption peaks corresponding to the thiol and epoxy groups. Although the epoxy peak exhibited a slight decrease of less than 7%, the thiol peak remained unchanged. These results indicate that any potential

reaction is negligible and likely falls within the range of experimental variation. Importantly, the actual printing time in this study is less than one hour, which is considerably shorter than the six-hour FTIR observation period. This further confirms that such reactions are unlikely to occur during the resin storage stage or the DLP printing process in this work.

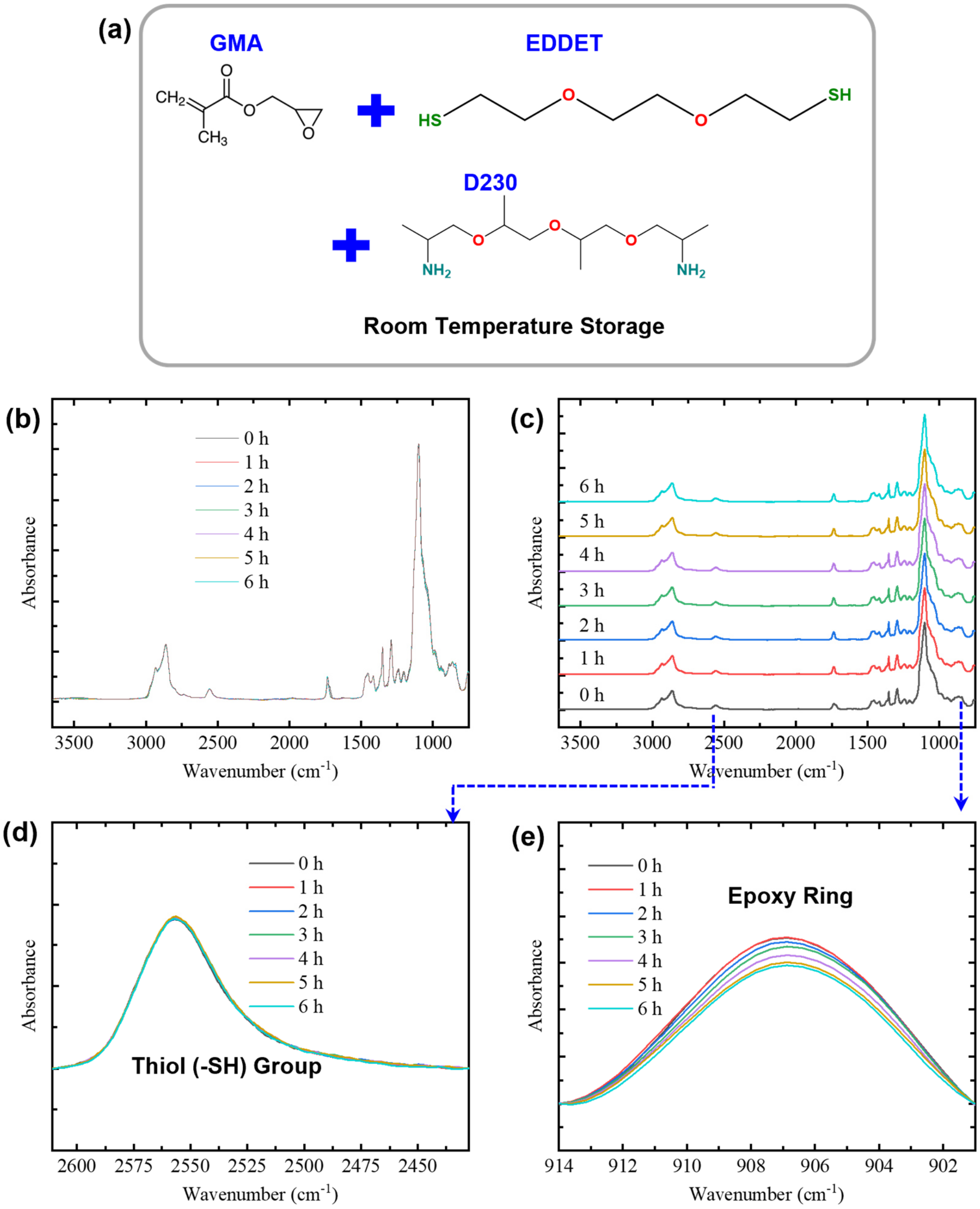

Figure S4. FTIR analysis of the model reaction between GMA, D230, and EDDET at room temperature. (a) Chemical structures of the reactants. (b) FTIR spectra in the range of 750–3500 cm^{-1}. (c) Vertically

offset spectra for clearer comparison. (d) Characteristic absorption peak of thiol group at 2556 cm^{-1}. (e) Characteristic epoxy ring absorption peak at 907 cm^{-1}.

Finally, the printable LCE resin containing all chemical components, including the catalysts and toluene solvent, was subjected to FTIR analysis after being stored at room temperature for various durations. As shown in Figures S5a and S5b, no observable changes were detected in any characteristic peaks, including those corresponding to the C=C bonds, thiol groups, and epoxy rings.

In a separate test, a precursor resin was prepared by mixing RM257, EDDET, and TPO at the same ratios as used in the printable ink. FTIR absorption spectra were recorded before and after heating at 80 °C for 30 minutes. This precursor mixture corresponds to an intermediate step in the ink preparation process, before mixing with epoxy content for DLP printing (see the Methods section of the main text). As shown in Figure S5c, the FTIR spectrum remains essentially unchanged, indicating that no notable chemical reactions occur during the 80 °C heating step. It is noted that, in contrast, if all components are mixed and then heated at 80 °C, substantial chemical reactions are expected because of the high reactivity between the amine, epoxy, and thiol.

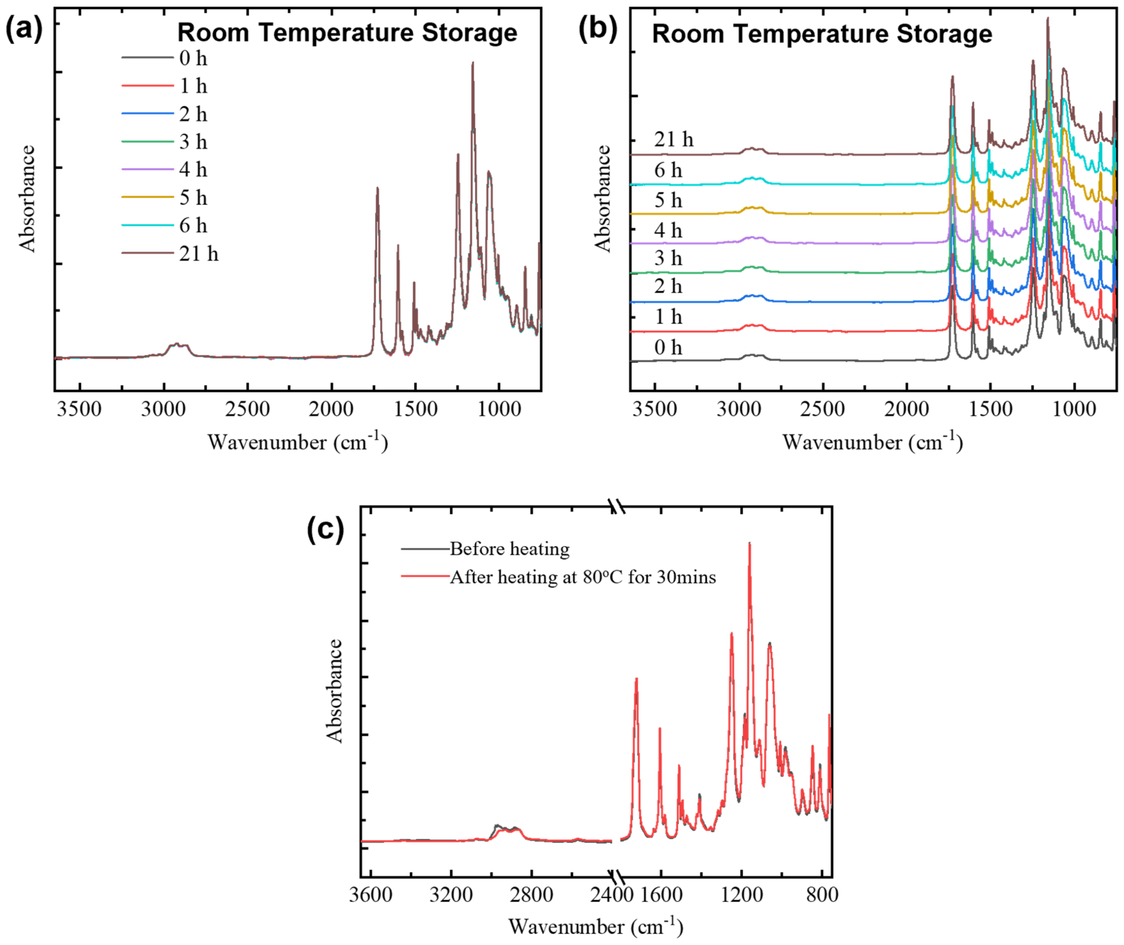

Figure S5. FTIR analysis of the printable LCE ink after (a, b) storage at room temperature and (c) heating at 80 °C for 30 min.

Overall, these FTIR characterizations confirm that during resin storage and during printing when the resin is not exposed to UV light, the chemical bonds within the printable LCE resin remain stable without any detectable reactions. Although certain reactions, such as epoxy–thiol and epoxy–amine reactions, are thermodynamically favorable, they do not occur under the conditions examined in this study. This stability is likely attributed to the presence of toluene in the printable ink. In this work, a moderate amount of toluene (30 wt%) was added to the ink to reduce its viscosity for DLP printing while avoiding noticeable volume shrinkage of the printed LCE after thermal treatment. The toluene solvent suppresses these unintended side reactions by diluting the reactive species and increasing the intermolecular distances between them, thereby enhancing the shelf life of the printable ink.

S3.2. Reactions during UV Polymerization in DLP Printing

To investigate the reaction mechanisms occurring during UV polymerization in DLP printing, a model reaction was first performed by mixing RM257, EDDET, and the photo-initiator TPO at the same molar ratios as used in the printable resin (Figure S6a). The mixture was irradiated using the same DLP projector with an exposure time of 15 seconds, which is identical to that used during printing.

Figure S6b presents the FTIR spectra before and after UV irradiation, and Figure S6c shows the spectral region of 1800–3600 cm^{-1} to highlight the relevant absorption peaks. Figures S6d and S6e display enlarged views of the peaks corresponding to the C=C bonds and thiol groups, respectively. The results show that the C=C peak nearly disappears after UV exposure, whereas the thiol peak decreases significantly but does not vanish completely. These observations indicate that, under the UV irradiation conditions used in DLP printing, the C=C bonds are more reactive than the thiol groups. This finding justifies the use of an excess amount of C=C bonds in the ink formulation relative to thiol groups, which avoids an excessive amount of unreacted thiol functionalities after printing.

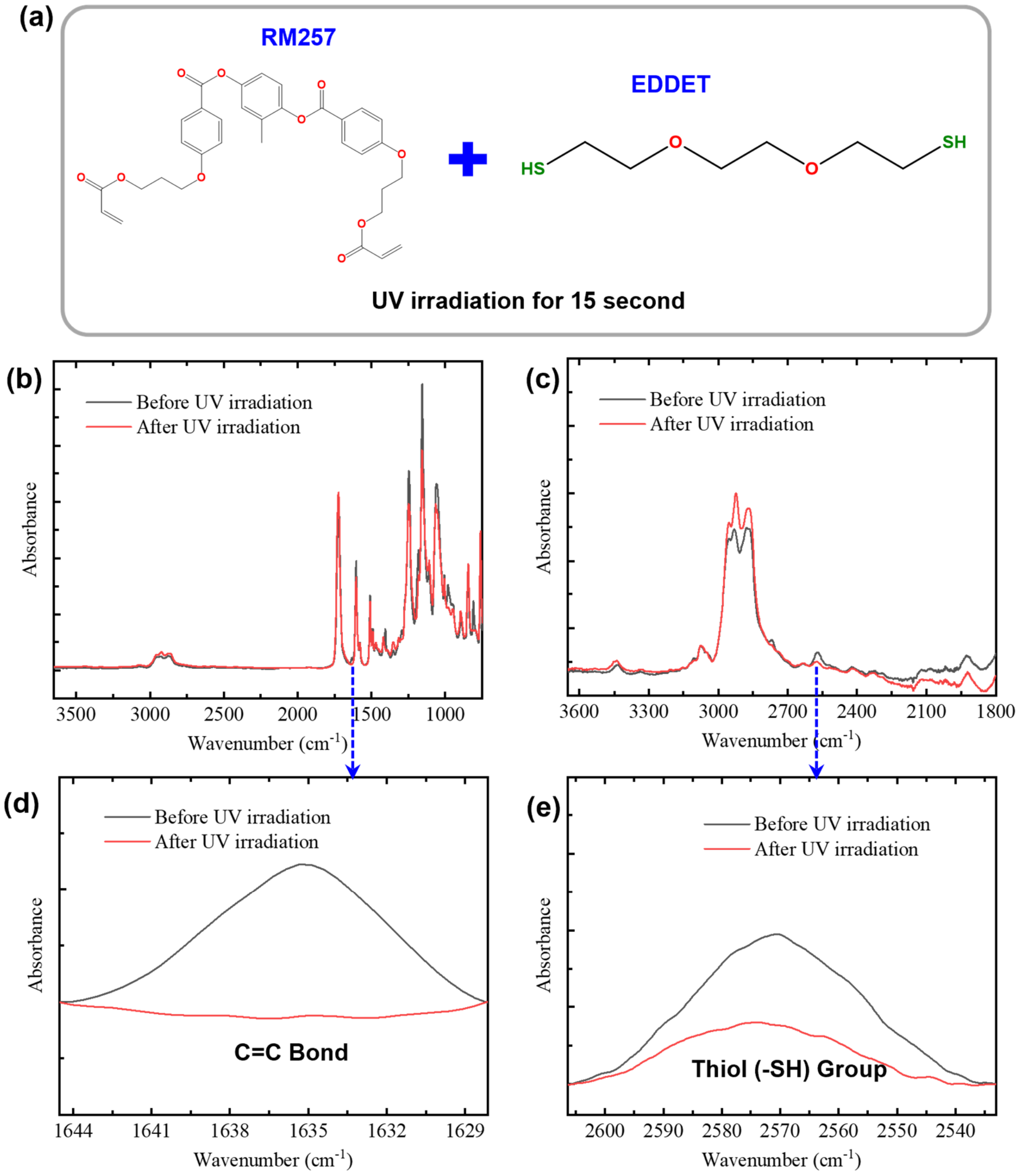

Figure S6. FTIR analysis of the model reaction between RM257 and EDDET with the addition of the photo-initiator TPO. (a) Chemical structures of RM257 and EDDET. (b) FTIR spectra in the range of 750–3500 cm^{-1} before and after UV irradiation. (c) FTIR spectra in the range of 1800–3600 cm^{-1} highlighting the relevant absorption peaks. (d) Characteristic absorption peak of C=C bonds. (e) Characteristic absorption peak of thiol groups.

To further confirm that the epoxy groups remain intact and are not attacked under free-radical conditions during UV exposure, another model reaction was performed by mixing GMA, EDDET, and the photo-initiator TPO (Figure S7a). This mixture simultaneously contained C=C, thiol, and

epoxy functional groups. The corresponding FTIR spectra (Figures S7b–e) display the absorption peaks associated with the C=C bonds, thiol groups, and epoxy rings. The results show that while the C=C bonds are completely consumed and the thiol peak decreases moderately, the epoxy peak at 907 cm⁻¹ remains unchanged.

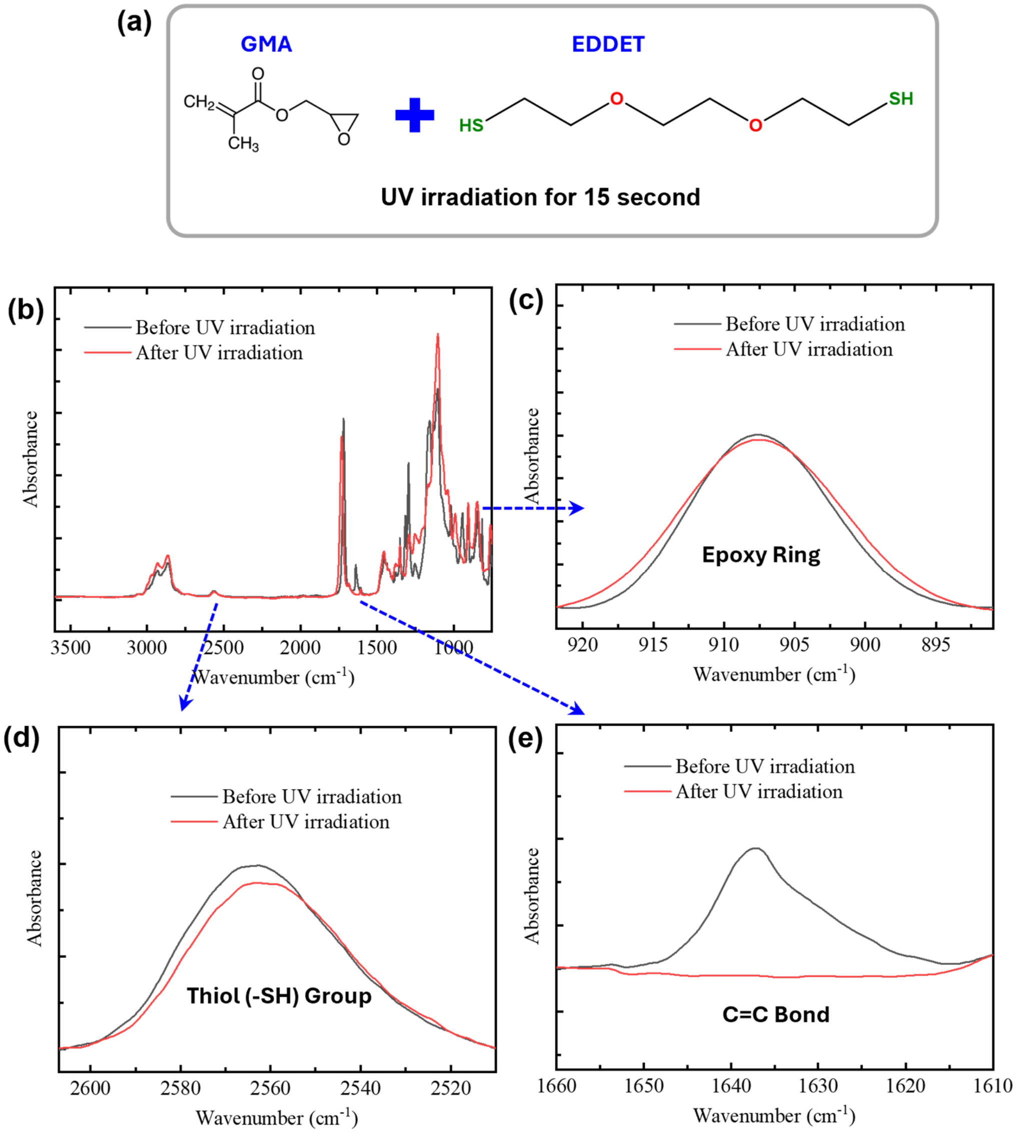

Figure S7. FTIR analysis of the model reaction between GMA and EDDET with the addition of the photo-initiator TPO. (a) Chemical structures of GMA and EDDET. (b) FTIR spectra in the range of 750–3500 cm⁻¹ before and after UV irradiation. Characteristic absorption peaks of (c) the epoxy ring, (d) thiol groups, and (e) C=C bonds.

These observations confirm that during the UV polymerization step of DLP printing, the reactions predominantly occur between the C=C bonds and thiol groups to form a loosely crosslinked LCE network. The epoxy groups remain chemically inert under these conditions and are fully preserved for subsequent post-curing reactions.

S3.3. Reactions during Thermal Treatment

To examine the chemical reactions occurring during the thermal treatment stage, a model reaction was conducted by mixing GMA and D230 and heating the mixture at 80 °C for various durations (Figure S8a). This temperature is identical to the thermal treatment temperature used for the printed LCE samples. Figures S8b and S8c present the full FTIR spectra at different heating times, while Figures S8d and S8e highlight the absorption peaks corresponding to hydroxyl (–OH) groups and epoxy rings, respectively. As heating time increases, the intensity of the epoxy peak decreases notably, whereas the hydroxyl (–OH) peak gradually increases as a reaction product. These observations confirm that an active ring-opening reaction occurs between the epoxy and amine groups at 80 °C.

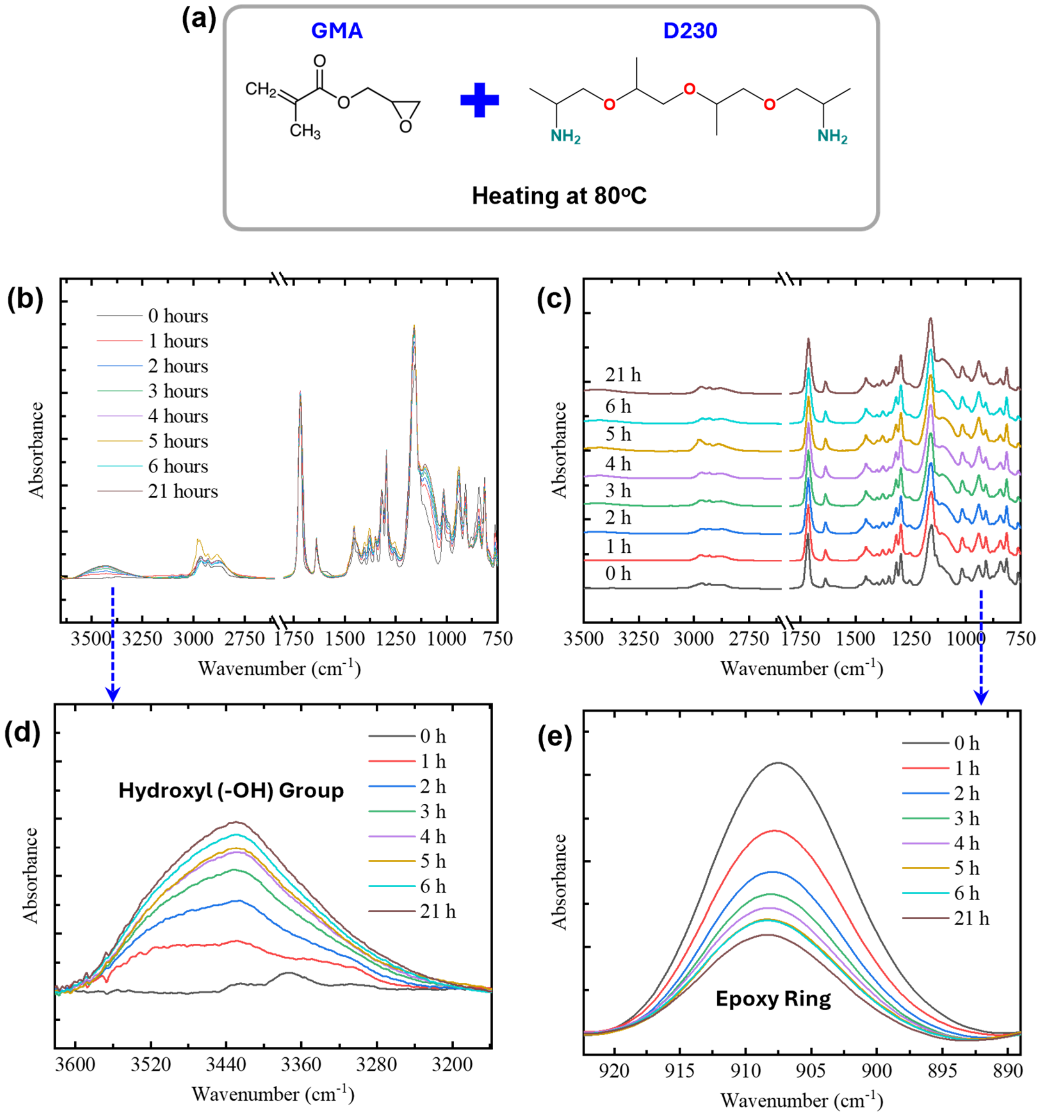

Figure S8. FTIR analysis of the model reaction between GMA and D230 at 80 °C. (a) Chemical structures of GMA and D230. (b) FTIR spectra in the range of 750–3500 cm^{-1}. (c) Vertically offset spectra for clearer comparison. Characteristic absorption peaks of (d) the hydroxyl group and (e) the epoxy ring.

S4. Programming and Shape Recovery of Two-stage LCE

Figure S9 illustrates the thermomechanical steps involved in the programming and actuation of conventional shape memory polymers (SMPs) and the developed two-stage LCE system.

For conventional SMPs (Figure S9a), the material is initially stiff because its glass transition temperature (T_g) is above room temperature (RT). To program the shape, the material is first heated above T_g, followed by the application of a global deformation. While maintaining this deformation,

the temperature is decreased back to RT and the load is then removed. Since the material is stiff at this point, it can largely retain the programmed deformation. During the recovery step, reheating the material above T_g activates polymer chain mobility, allowing the structure to return to its original shape due to entropic elasticity. It is important to note that this shape change is one-way; to induce further shape change, the material must be reprogrammed.

For the two-stage LCE system (Figure S9b), the material is soft at RT and thus can be directly applied with a global programming deformation. While maintaining this deformation, the temperature is increased above the epoxy curing temperature (T_e) and held for a prescribed duration. This allows epoxy linkages to gradually form and fix the deformation. After curing, the external load can be removed, and the programmed shape is retained. During actuation, the material reversibly transitions between the programmed shape and the original 3D-printed configuration as the temperature cycles across the phase transition temperature (T_i).

Overall, the programming and actuation procedures of the developed LCE system are simple and user-friendly. Similar to conventional SMPs, the material can be programmed and activated through basic mechanical deformation and heating. However, due to its distinct network architecture, the LCE system ultimately exhibits reversible actuation behavior.

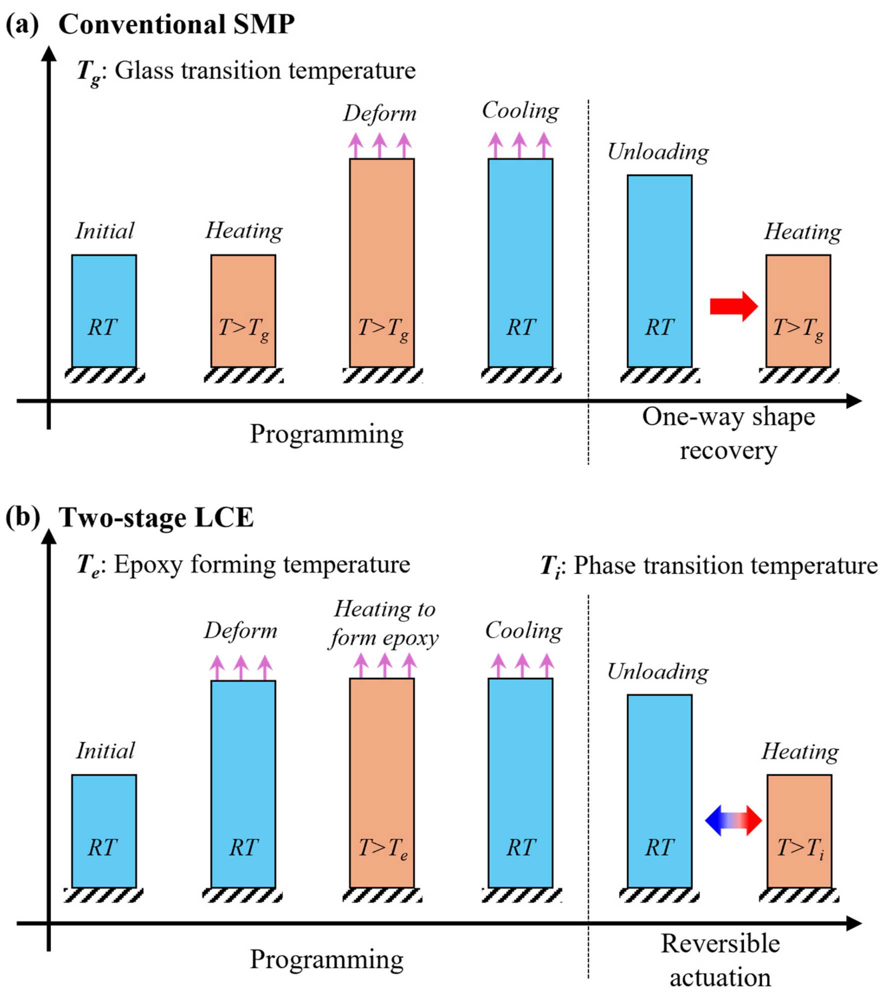

Figure S9. Schematic view showing the thermomechanical steps involved in the programming and actuation stages. (a) Conventional SMPs. (b) The developed two-stage LCE system.

S5. Resin Compositions for LCE 4D Printing

The detailed compositions of each ink formulation for LCE samples are provided in Table S1. For all ink formulations, the molar ratio between RM257 and EDDET was fixed at 1:0.87, and the molar ratio between GMA and D230 was fixed at 1:0.18. However, the overall molar ratio between the acrylate and epoxy networks varied across samples, ranging from 1:0 to 1:0.8.

Table S1. Molar Content of Precursor Resins when printing LCE samples using DLP

**Sample Label**	**Molar Content of Precursor Resins (Normalized to RM257)**	
	Acrylate network	Epoxy network

	RM257	EDDET	GMA	D230
**AE_0**	1.00	0.87	0	0
**AE_1**	1.00	0.87	0.01	0.18×10^{-2}
**AE_3**	1.00	0.87	0.03	0.54×10^{-2}
**AE_6**	1.00	0.87	0.06	1.08×10^{-2}
**AE_12**	1.00	0.87	0.12	2.16×10^{-2}
**AE_18**	1.00	0.87	0.18	3.24×10^{-2}
**AE_30**	1.00	0.87	0.30	5.40×10^{-2}
**AE_80**	1.00	0.87	0.80	14.40×10^{-2}

S6. Thermomechanical Properties of LCEs

Figure S10 presents the thermomechanical properties of the LCE samples (AE_0 to AE_80), including their storage moduli, tan δ, and room-temperature stress–strain curves. As shown by the DMA data in Figures S10a, S10b, S10d, and S10e, samples AE_0, AE_6, AE_12, and AE_18 exhibit similar storage moduli and consistent glass transition temperatures (Tg) around 10 °C before and after thermal treatment. A second peak is observed in the their tan δ curves, which corresponds to the mesogen phase transition temperature. Before thermal treatment, this transition appears between 35–50 °C and tends to decrease with increasing epoxy content, possibly due to a reduced mesogen fraction. After thermal treatment, the mesogen phase transition temperature increases to the range of 50–65 °C.

In contrast, AE_30 and AE_80 exhibit higher storage moduli, which further increase after thermal treatment. This enhancement is attributed to the substantial epoxy network content, which significantly increases the network crosslinking density. For the same reason, the Tg of AE_30 and AE_80 increases above room temperature, indicating stiffer materials suitable for load-bearing applications. Notably, no second peak is observed in the tan δ curves for these two samples, likely due to the lower mesogen content resulting from the higher epoxy concentration.

Figures S10c and S10f show the room-temperature stress–strain responses before and after thermal treatment. Samples AE_0, AE_6, AE_12, and AE_18 display similar mechanical behavior prior to thermal treatment, with tensile strengths around 1 MPa and stretchability of approximately 2.5. Thermal treatment slightly improves both stretchability and strength. In contrast, AE_30 and AE_80 exhibit markedly higher moduli and strength after thermal treatment. Specifically, AE_80 demonstrates more than a threefold increase in tensile strength and approximately a 20-fold

increase in stiffness.

Overall, the comparison suggests that incorporating a moderate amount of epoxy (less than 30%) does not significantly alter the thermomechanical properties or phase transitions of the LCE samples, allowing them to remain suitable for soft actuator applications. However, increasing the epoxy content above 30% substantially enhances material stiffness, making the LCEs more appropriate for load-bearing applications, although at the expense of reduced actuation strain.

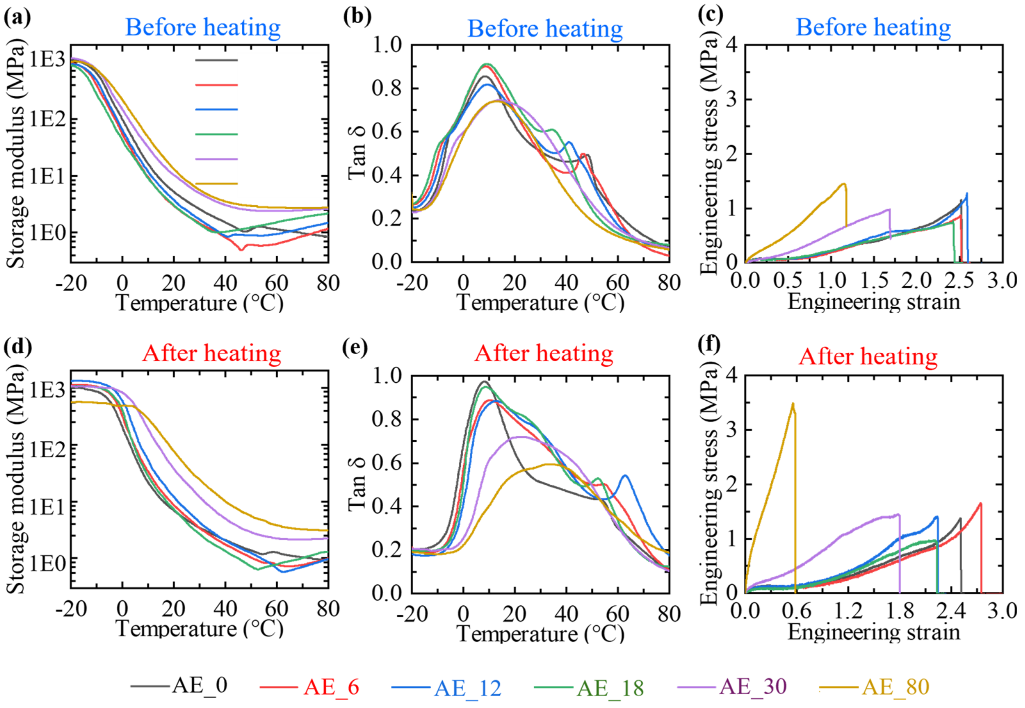

Figure S10. Thermomechanical properties of LCE samples AE_0 to AE_80. (a) Storage moduli before thermal treatment. (b) Tan δ before thermal treatment. (c) Stress-strain curves before thermal treatment. (d) Storage moduli after thermal treatment. (e) Tan δ after thermal treatment. (f) Stress-strain curves after thermal treatment.

S7. Actuation Strain Characterization

The actuation strain of printed LCE samples with varying molar ratios of epoxy network was characterized by tracking the distance between two marked points during a heating–cooling cycle. As shown in Figure S11a, all samples, with the same initial length of 20 mm, were uniformly stretched to 80% engineering strain and then programmed at 80 °C for 14 hours. After unloading at room temperature, the distance between the two marked points was recorded, as shown in Figure

S11b.

Upon heating to 100 °C, the samples tended to recover toward their as-printed geometries, resulting in a reduction in the distance between the marking points (Figure S11c). The actuation strain was calculated as the decrease in length divided by the initial length of 20 mm. The data is reported in Figure 3d of the main manuscript.

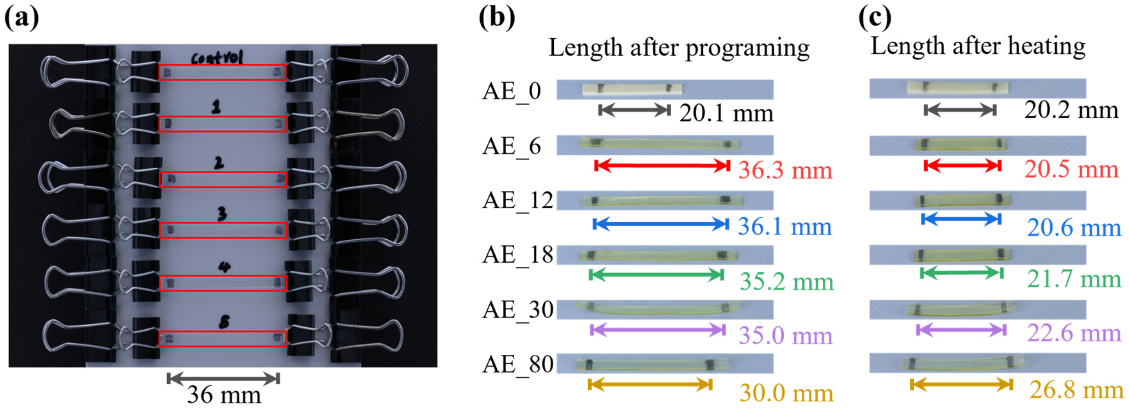

Figure S11. Distance between two marked points during a heating–cooling cycle. (a) Pictures of the sample. (b) Length after programming. (c) Length after heating.

S8. Polarized Fourier-Transform Infrared Spectroscopy Measurements

Polarized Fourier-Transform Infrared Spectroscopy (FTIR) was used to evaluate the alignment of mesogens in the LCE samples after the second-stage programming. Measurements were performed using a Nicolet iS50 FTIR spectrometer (Thermo Fisher Scientific, Waltham, MA, USA) in transmission mode, with sample thickness maintained below 0.5 mm. The instrument is equipped with a built-in ZnSe polarizer that can rotate in 1° increments up to 180°.

Figure S12 presents the FTIR spectra of various LCE samples, with light polarization oriented either parallel or perpendicular to the mesogen alignment direction. During the measurements, infrared light passed through the sample and excited specific vibrational modes (e.g., stretching or bending) of functional groups, which appeared as absorption peaks in the spectrum. The C–H stretching mode exhibited the highest absorption when the polarization was perpendicular to the mesogen alignment, because the electric field vector of the light aligned with the bond axis. Conversely, the lowest absorption occurred when the polarization was parallel to the mesogen alignment. The C–H bending vibrations demonstrated the opposite trend in absorption intensity.

The polarization angle of the incident light was then rotated from 0° to 180° in 10° increments. At each angle, FTIR spectra were recorded in under 2 seconds. The absorption peak of the C–H stretching vibration was monitored and is summarized in Figure S13 as a function of polarization angle (blue dots). The differences in the absorption peaks are used to identify the mesogen order parameter, S. Specifically, the maximum and minimum absorption peaks of the C-H bonds, A_{max} and A_{min}, are used to calculate the dichroic ratio of the anisotropic LCE network as $D = A_{max}/A_{min}$. The order parameter, S, is determined as $S = (D - 1)/(D + 2)$. For the AE_0 sample, which contains no epoxy, the LCE remains in a polydomain state after programming, meaning the mesogens are not aligned. As a result, the FTIR absorption peaks are nearly identical in all polarization angles. Therefore, D approaches one, and S approaches zero.

The data of the mesogen order parameter is reported in Figure 2e of the main manuscript.

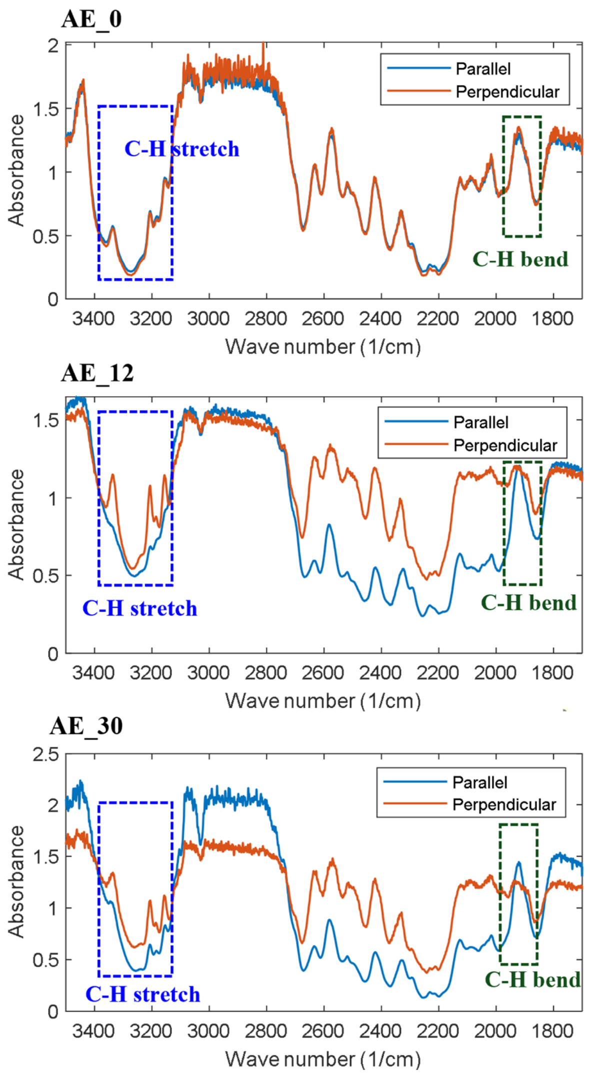

Figure S12. FTIR spectra of LCE samples with varying epoxy content, measured under parallel and perpendicular light polarization relative to the mesogen alignment.

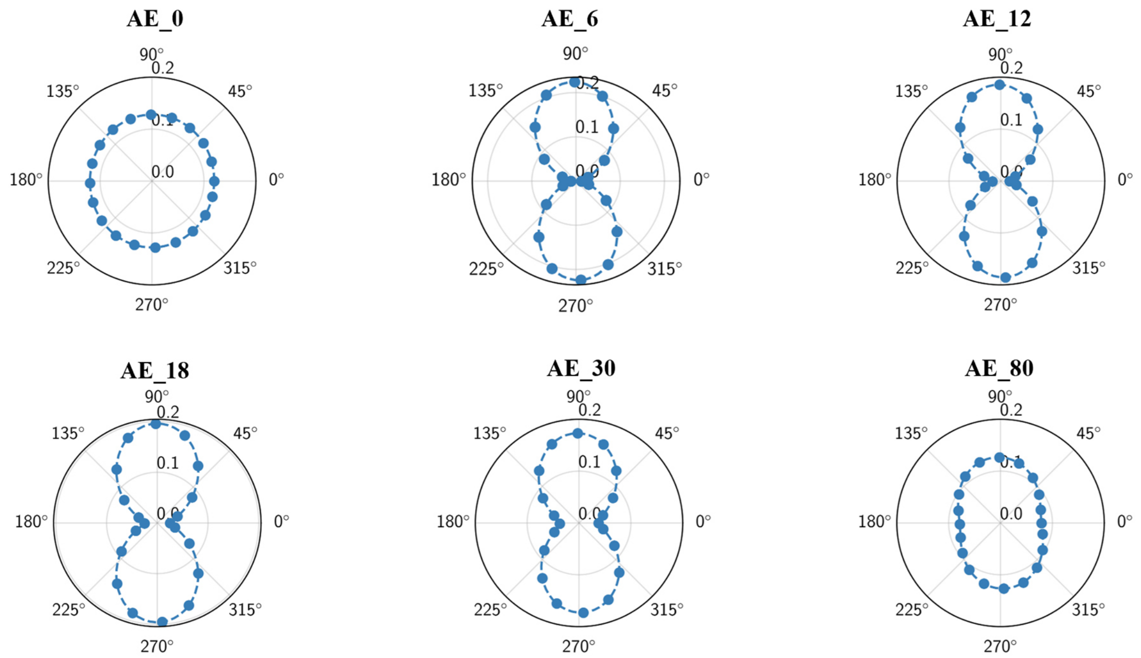

Figure S13. Polar plots of C–H stretching absorption intensity as a function of light polarization angle for different LCE samples.

S9. FEA Simulations of Reversible Folding Deformation

Finite Element Analysis (FEA) simulations were conducted to investigate the relationship between reversible axial actuation and folding deformation in LCE structures.

First, the axial actuation strain of printed AE_6 LCE samples was characterized as a function of programming strain, which ranged from 1% to 80%. The corresponding actuation strain was measured following the method described in Section S4 and is summarized in Figure S14. The results show that when the programming strain is small (e.g., below 5%), the actuation strain is negligible. However, beyond a certain threshold (8%), the actuation strain becomes significant and even exceeds the programming strain.

This phenomenon occurs because, at lower programming strains, the mesogens are not fully aligned along the programming direction. Upon unloading at room temperature, increased mesogen–mesogen interactions lead to their further alignment and additional elongation of the sample, such that the sample length after unloading is greater than the displacement imposed during programming. As a result, the measured actuation strain exceeds the applied programming strain.

When the programming strain exceeds 60%, it is sufficiently large to induce full alignment of

the mesogens. In this regime, the actuation strain becomes approximately equal to the programming strain.

Based on these experimental observations, a fourth-order polynomial function was used to fit the actuation strain data (ε_r), shown as the red curve in Figure S5:

$$\varepsilon_r(\varepsilon_p) = \begin{cases} -8.2\varepsilon_p^4 + 19.7\varepsilon_p^3 - 16.3\varepsilon_p^2 + 5.9\varepsilon_p - 0.2 & if\ \varepsilon_p > 0.05 \\ 0 & if\ \varepsilon_p < 0.05 \end{cases} \quad \text{(S1)}$$

where in ε_p is the programming strain.

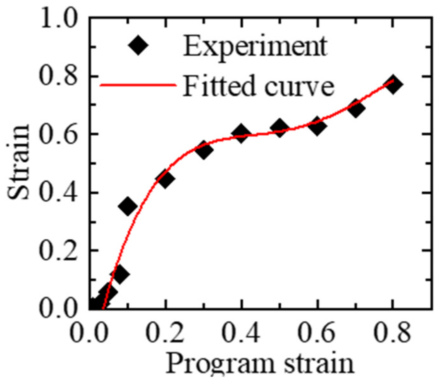

Figure S14. Axial actuation strain of printed AE_6 LCE sample as a function of the programming strain.

Subsequently, the reversible folding deformation of the printed LCE strip was characterized. During programming, samples with a uniform thickness of 0.7 mm were folded and placed inside a hollow cylindrical mold, with their length precisely matching the circumference of the cylinder. The samples were then heated at 80 °C for 14 hours and removed from the mold at room temperature. Figure S15a shows the appearance of samples programmed with different radii of curvature. It is observed that when the programming curvature exceeds 0.35 mm^{-1}, the samples can fully retain the curved configuration. Below this critical curvature, the shape fixity decreases, as evidenced by the incomplete circular profiles.

An FEA model was developed in ABAQUS to predict the folding deformation of the LCE strip. The model consisted of a 0.7 mm-thick strip discretized into six bending layers (Figure 2h). The mid-plane was defined as the neutral surface. During the folding programming process, the top three layers experienced compressive strain, while the bottom three layers were subjected to tensile strain. The axial strain in each layer was estimated using the relation $\varepsilon_p = -y/R$, where y is the distance from the neutral surface, and R is the folding radius.

The programming strains for each bending layer (ε_p) were then substituted into Eq. S1 to calculate their axial actuation strains (ε_r). For the compressive layers, the axial compressive programming strain was first used to determine the corresponding transverse tensile strain under the assumption of incompressible LCE. This transverse programming strain was then input into Eq. S1 to calculate the transverse actuation strain, which was subsequently converted into the axial actuation strain for the compressive layers.

After determining the actuation strains, directional thermal expansion coefficients were assigned to each layer as:

$$\alpha=\varepsilon_r/\Delta T, \tag{S2}$$

where ΔT is the temperature changes during actuation, from room temperature to 100 °C. This modeling approach estimates the final folding deformation but does not simulate the evolution of curvature during heating.

As shown in Figure S15b, the predicted folding curvature aligns well with experimental observations. For programming curvatures below 0.35 mm^{-1}, the samples exhibit lower shape fixity. This is because the central layers near the neutral surface develop axial strains below 5%, which is the threshold for notable actuation strain as identified in Figure S14. These layers thus contribute little to the folding actuation and resist deformation imposed by the other active bending layers. In contrast, when the programming curvature exceeds 0.35 mm^{-1}, the predicted actuation curvature closely matches the programmed curvature.

Overall, the agreement between FEA predictions and experimental results confirms that folding deformation can be fundamentally interpreted as a distributed form of axial actuation across the bending layers.

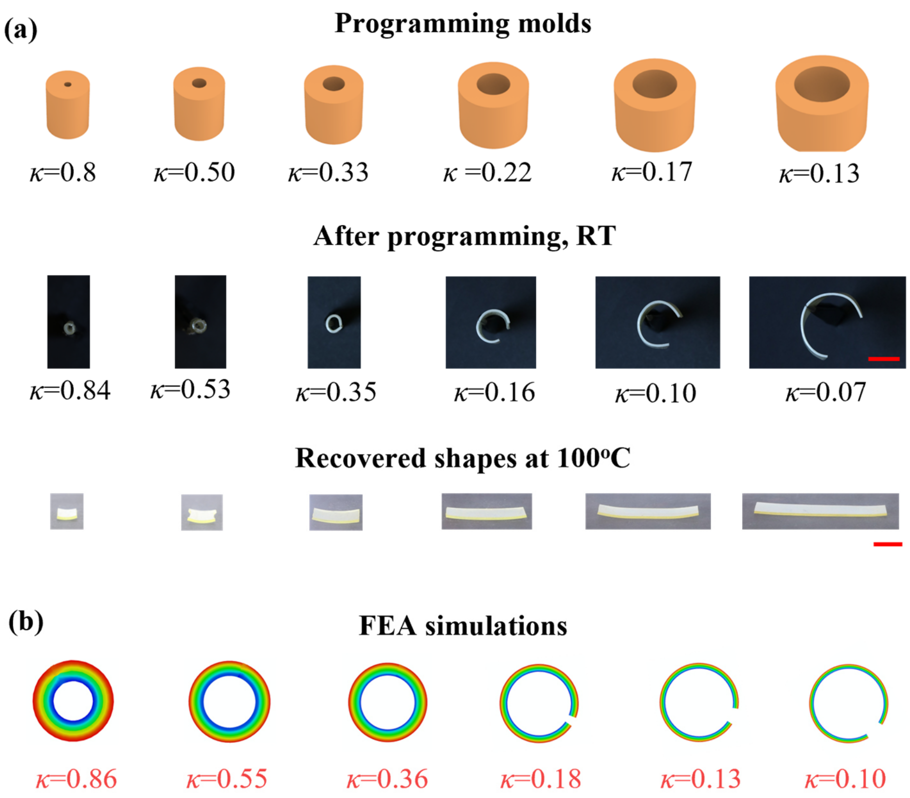

Figure S15. Experimental and simulation results for the folding programmability and shape recovery behavior of LCE strips. (a) Experimental results showing the folding deformation of LCE strips programmed with different curvatures. (b) FEA simulation results of the folding deformation, demonstrating strong agreement with experimental observations across various programmed curvatures. Scale bar = 1cm.

S10. FEA Simulation of Lattice Metamaterial

In Figure 3b of the manuscript, the stiffness of the lattice metamaterial is evaluated under different strain levels. Herein, we want to examine the stiffness of the lattice without internal stress, as if they are newly fabricated structure. Therefore, it is not appropriate to continuously compress a single lattice structure and directly extract stiffness values, as residual stresses from prior deformations would affect the results.

To accurately measure the stiffness as if the lattice were newly fabricated and free from deformation history, each simulation begins with a reconstructed lattice geometry that reflects the targeted strain level. The initial lattice structure is generated using the same CAD model employed for 3D printing. It is first compressed to the desired strain level (e.g., 15%), and the corresponding

stiffness is calculated by dividing the global stress by the global strain. The nodal displacements from this step are recorded, and the updated node positions (original positions plus displacements) are used to redefine the mesh for the next simulation step. This process ensures that each simulation starts from a stress-free configuration corresponding to the pre-compressed geometry.

The updated lattice model is then subjected to a small additional compression to determine the next stiffness point, and the process is repeated. This stepwise reconstruction guarantees that stiffness is evaluated under a consistent, unloaded initial condition. Figure S16 shows examples of the reconstructed lattice models at different pre-compression stages. Each model contains approximately 660,000 tetrahedral elements.

In this study, the FEA simulations were performed using the AE_6 sample. The material was modeled as a linear elastic solid. For simulations conducted at room temperature, such as those examining the auxetic metamaterials, a constant elastic modulus of 1.05 MPa and a Poisson's ratio of 0.49 were used. The modulus was obtained from uniaxial tensile tests of the AE_6 sample after thermal treatment, as presented in Figure 11 of the main text.

For simulations involving temperature variations, the modulus values at different temperatures were derived from DMA data. As shown in Figure S10d, the modulus of the AE_6 sample after thermal treatment decreases slightly from ~3 MPa at room temperature to ~1 MPa at 80 °C. It should be noted that the modulus measured by DMA at room temperature differs slightly from that obtained by uniaxial tension, which is expected due to differences in the testing methods. To maintain consistency across all FEA simulations, the room temperature modulus obtained from the uniaxial tensile test (1.05 MPa) was used as the reference value. The temperature-dependent modulus from DMA was then applied to scale this reference modulus. Specifically, the modulus used in the FEA model at each temperature was obtained by multiplying the tensile test modulus by the ratio of the DMA-measured modulus at that temperature to the DMA-measured modulus at room temperature. The resulting temperature-dependent modulus data were input into ABAQUS as a temperature-dependent property. This approach ensures that the simulations consistently reflect the experimentally observed stiffness variation with temperature while maintaining a unified reference modulus.

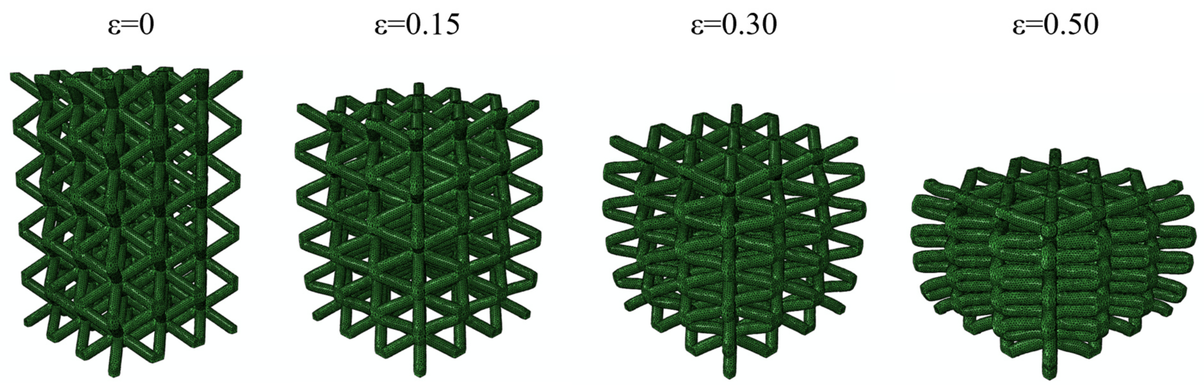

Figure S16. Reconstructed lattice models at different pre-compression stages.

S11. Unit Cell Shape Change of Bistable Metamaterials

Figure S17a shows the as-fabricated bistable metamaterials used in the characterization presented in Figures 3g–4j. Demonstrations in the manuscript reveal that, upon heating, the LCE strip can overcome the energy barrier between State I and State II and fully recover its original configuration. However, this recovery capability depends on the programming configuration, which determines both the actuation strain and actuation force of the LCE strip.

To illustrate this dependence, sine-curved LCE strips with different amplitudes (a = 5 mm and a = 7 mm) were fabricated, as shown in Figure S17b. After programming, both samples were subjected to heating. Only the sample with the smaller amplitude (a = 5 mm) was able to fully transition to State II with successful shape recovery. This behavior is also demonstrated in Supplementary Video 3. These comparisons raise intriguing questions for future investigations regarding the reversibility and performance of bistable metamaterials as functions of both material properties and geometric design parameters.

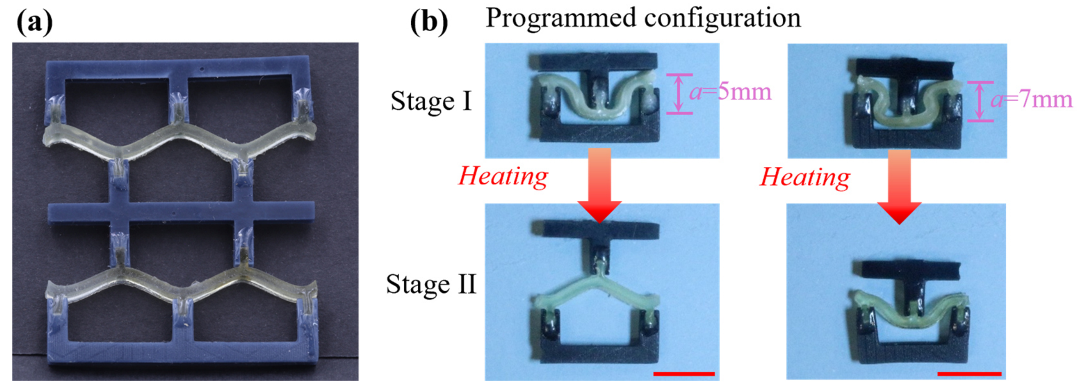

Figure S17. Unit Cell Shape Change of Bistable Metamaterials. (a) As-fabricated bistable metamaterials.

(b) Shape recovery of unit cells with different programming displacements. Scale bar = 1cm.

S12. Inchworm-like crawling soft robot

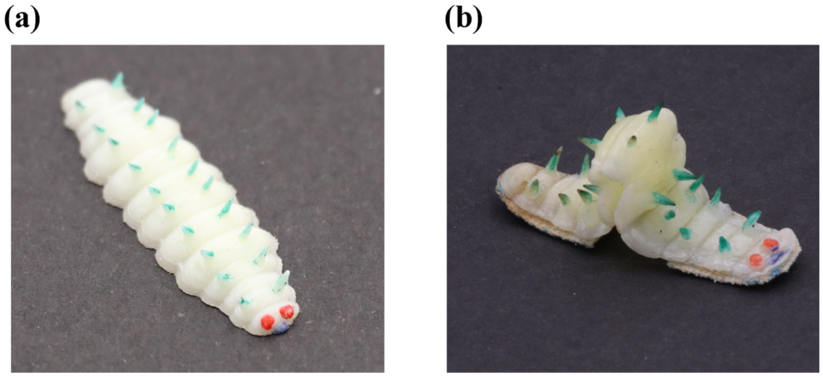

Figure S18. 4D-printed inchworm-like crawling soft robot. (a) As-printed configuration before programming. (b) Room-temperature configuration after programming.
