# Supplementary material for: 4D printing through vat photopolymerization of two-stage UV-curable liquid crystal elastomers
Source: Nat Commun. 2026 Jan 15;17:1671. doi: 10.1038/s41467-026-68370-y (PMC12910083; doi:10.1038/s41467-026-68370-y)
Supplement: Supplementary file 2 — Description of Additional Supplementary Files [file 41467_2026_68370_MOESM2_ESM.pdf]

Description of Additional Supplementary Files

Supplementary Movie 1. Reversible shape change of a printed LCE auxetic metamaterial.

Supplementary Movie 2. Reversible shape change of a printed bistable metamaterial unit cell.

Supplementary Movie 3. Shape recovery of printed bistable metamaterials programmed with different displacement.

Supplementary Movie 4. Reversible shape changes of a printed LCE deployable antenna.

Supplementary Movie 5. Reversible shape changes of a printed LCE morphing aircraft model.

Supplementary Movie 6. Reversible shape changes of a printed LCE Eiffel Tower model.

Supplementary Movie 7. Reversible shape changes of a printed LCE vascular stent.

Supplementary Movie 8. Reversible shape changes of a printed LCE single-layer flower model.

Supplementary Movie 9. Reversible shape changes of a printed LCE double-layer flower model.

Supplementary Movie 10. Reversible shape changes of a printed LCE hand model showing the OK gesture.

Supplementary Movie 11. Reversible shape changes of a printed LCE hand model showing the rock and roll gesture.

Supplementary Movie 12. Reversible shape changes of a 3D printed prosthetic arm.

Supplementary Movie 13. A simplified soft robotic system that grips and lifts a dumbbell.

Supplementary Movie 14. Inchworm-like crawling motion under successive heating and cooling cycles
