# Supplementary material for: 4D printing through vat photopolymerization of two-stage UV-curable liquid crystal elastomers
Source: Nat Commun. 2026 Jan 15;17:1671. doi: 10.1038/s41467-026-68370-y (PMC12910083; doi:10.1038/s41467-026-68370-y)
Supplement: Supplementary file 17 — Source Data [file 41467_2026_68370_MOESM17_ESM.pdf]

nature portfolio

Peer Review File

4D Printing through Vat Photopolymerization of Two-Stage UV-Curable Liquid Crystal Elastomers

Corresponding Author: Dr Kai Yu

This file contains all reviewer reports in order by version, followed by all author rebuttals in order by version.

Version 0:

Reviewer comments:

Reviewer #1

(Remarks to the Author)
Title: 4D Printing through Vat Photopolymerization of Two-Stage UV-Curable Liquid Crystal Elastomers
Authors: Kai Yu et al.
The authors report an interesting system for 4D printing using a DLP setup with a two-stage curing process: UV curing for acrylate and thiol groups, followed by thermal curing of epoxy moieties. They also demonstrate the applicability of their system through numerous examples. However, several modifications are necessary to support their claims. Below are my detailed comments:
1. A more detailed analysis of the acrylate and thiol groups is necessary to substantiate the proposed two-stage curing mechanism.
2. The structure during the first curing stage may correspond to a polydomain nematic state, which is not discussed in the manuscript. The actuated state at high temperature likely corresponds to the isotropic phase. These two states differ at the molecular level, and the recovered state at 100 °C may not replicate the first-stage state. The authors should provide an explanation of this, preferably with actual schematics—Figures 1d to 1h do not accurately represent the liquid crystalline state.
3. Figure 2b does not show the actuation speed of the LCE specimens, despite the statement: “While Figure 2b compares the actuation speeds of LCE specimens over a fixed temperature range.” Although actuation speed is indirectly represented in the cyclic test, this is not an appropriate way to discuss or quantify it.
4. The discussion in lines 198 to 226 is confusing and lacks clarity. The authors should revise this section to present their claims in a more structured and coherent manner.
5. Some figures are explained out of order, which disrupts the logical flow of the manuscript.

Reviewer #2

(Remarks to the Author)
1. This manuscript presents many interesting results and images. However, from a chemical standpoint, as shown in Figure 1(b), the monomer mixture consists of diacrylate, dithiol, diamine, and epoxy monomers. In the first stage, the dithiol and diacrylate undergo free-radical polymerization, while the epoxy groups are expected to react simultaneously in the presence of diamine. Therefore, the reaction pathway may not match the one described in Figures 1(d) and (f). The authors should provide FTIR evidence to demonstrate the evolution of functional groups throughout the entire programming process. Furthermore, since epoxy groups (GMA) can undergo ring-opening reactions under free-radical conditions, the authors must explain how the epoxy rings are preserved during the initial polymerization stage. Meanwhile, the acrylate–thiol Michael addition proceeds in the presence of basic amines.
2. As shown in Table S1, the overall molar ratio between the acrylate and epoxy networks varies across the samples, ranging from 1:0 to 1:0.8. Under these conditions, the excess diacrylate would also undergo free-radical polymerization during the first reaction stage. Given this, what is the rationale for incorporating an excess amount of diacrylate? If the programmed polymerization pathway differs from that illustrated in Figures 1(d) and (f), the validity of the results presented in this manuscript becomes questionable.
3. The proposed two-stage polymerization is designed based on the stoichiometry of functional groups. However, from a chemical standpoint, the feasibility of the designed functional group reactions is questionable. As this issue is central to the manuscript, it must be thoroughly clarified before the manuscript can be considered for further review.

4. As described in the experimental section, RM257, GMA, EDDET, and D230 were mixed and stored at room temperature in the dark for 12 hours. Theoretically, in the presence of amines, acrylate–thiol Michael addition should proceed even at room temperature. How, then, is the occurrence of this reaction controlled?
5. This manuscript presents many interesting results and images based on the proposed two-stage polymerization. However, from a theoretical standpoint, the feasibility of the controlled two-stage polymerization for the designed monomer mixtures is questionable. Spectral evidence supporting the proposed reactions at each stage must be provided. How are the functional groups controlled during the process? How is the epoxy ring prevented from opening in the presence of basic amines or under free-radical polymerization conditions? What mechanisms ensure that epoxy ring opening does not occur during free-radical reactions? Furthermore, why does excess acrylate not undergo polymerization during the free-radical stage?
6. During the thermal treatment, the authors mentioned that LCE materials tended to align along the local stress directions, resulting in a monodomain in the LCE. It is recommended to explain the phenomenon in a more detailed way with the support of schematic illustrations or further experimental results.

There are numerous critical issues that require clarification. The manuscript must undergo “major revisions” to address these problems before it can be considered for further review.

Reviewer #3

(Remarks to the Author)
The manuscript presents a 4D printing method for liquid crystal elastomers (LCEs) using vat photopolymerization via a two-stage curing process. This newly developed approach enables high-resolution 4D printing of LCEs, representing a significant advancement for engineering applications. The manuscript is well organized. However, several questions remain to be addressed.
1. Compared to direct ink writing (DIW), the DLP method offers a significant advantage in resolution. It is suggested to include the printing resolution data and add the comparison in the manuscript.
2. Two-stage polymerization process represents the most widely used strategy for 4D printing of LCEs. The critical aspect lies in the distinct reaction rates or reaction conditions between the two polymerization stages. Otherwise, it becomes challenging to achieve the reproducible programming of LCEs. It is known that thiol-epoxy reaction can also happen in the ambient condition. How about the reaction kinetics of the thiol-acrylate? Since the 3D printing time for the objects in different sizes is different significantly. It is important to study the reaction kinetics of thiol-epoxy reaction.
3. The curing temperature for the amine-epoxy is higher than the phase transition temperature of 3D printed elastomer. At this higher temperature, the LCEs before thermal curing is in the isotropic state. Will this affect the aligning process?
4. On page 6, the authors said that “A relatively low processing temperature of 80 °C was selected to minimize the risk of thermal degradation”. It may be convincible that the TGA results are provided. Common polymeric materials typically undergo thermal degradation above 300 C. Compared to the degradation temperature, the phase transition temperature of the firstly crosslinked LCEs represents a more critical parameter for the second curing process.
5. Compression programming is employed to configure the actuation behavior of 3D-printed LCE metamaterials. However, the initially crosslinked LCE network typically exhibits low mechanical stiffness, raising concerns about structural instability during compression. How to solve this problem?
6. How about the parameters used in the FEA simulations? The mechanical parameters should be measured in appropriate temperatures.
7. Polyphenylene oxide (PPO) is conventionally known as a high-performance engineering polymer, not a photoinitiator. Please check it.
8. On Page 5, the authors said that “This complete shape fixity was attributed to the ring-opening polymerization between GMA and the unreacted D230”. The “ring-opening polymerization” usually refers to a type of chain-growth polymerization where cyclic monomers are attacked by the terminus of a polymer chain, leading to the formation of longer polymer chains. The amine-epoxy reaction here is a ring-opening reaction instead of ring-opening polymerization.

Version 1:

Reviewer comments:

Reviewer #1

(Remarks to the Author)
Title: 4D Printing through Vat Photopolymerization of Two-Stage UV-Curable Liquid Crystal Elastomers
Authors: Kai Yu et al.

The revised manuscript satisfactorily addresses all of my previous comments. The authors have appropriately clarified the experimental methodology, improved the mechanistic discussion, and strengthened the supporting data where necessary. I find the revisions comprehensive and acceptable. Therefore, I have no further concerns and recommend the manuscript for publication in its current form.

Reviewer #2

(Remarks to the Author)

All the raised questions are resolved, and I have no further questions about this manuscript.

Reviewer #3

(Remarks to the Author)
The authors have thoroughly addressed the major concerns. The manuscript is significantly improved. I recommend acceptance of the manuscript after minor revision. Minor suggestions:
1) It is very interesting that the printable ink, which contains highly reactive components (amine catalysts, thiol, and epoxy groups), remains stable even after being heated to 80 °C for 30 minutes. This exceptional stability is highly counterintuitive considering the known rapid reaction kinetics of thiol-epoxy systems in the presence of amine catalysts. To validate this claim, I strongly recommend the authors provide additional supporting evidence, for instance, performing 1H NMR spectroscopy on the ink composition before and after treatment.
2) Regarding the authors' response to Comment 4 from Reviewer 3, a clarification on the programming temperature is warranted. It is not strictly necessary for the programming temperature to exceed the phase transition temperature. The chain mobility is sufficient enough when temperature is higher than the glassy transition temperature.

Version 2:

Reviewer comments:

Reviewer #3

(Remarks to the Author)
All my concerns have been clarified by the authors. I recommend acceptance of the manuscript.

Open Access This Peer Review File is licensed under a Creative Commons Attribution 4.0 International License, which permits use, sharing, adaptation, distribution and reproduction in any medium or format, as long as you give appropriate credit to the original author(s) and the source, provide a link to the Creative Commons license, and indicate if changes were made.
In cases where reviewers are anonymous, credit should be given to 'Anonymous Referee' and the source.
The images or other third party material in this Peer Review File are included in the article's Creative Commons license, unless indicated otherwise in a credit line to the material. If material is not included in the article's Creative Commons license and your intended use is not permitted by statutory regulation or exceeds the permitted use, you will need to obtain permission directly from the copyright holder.
To view a copy of this license, visit https://creativecommons.org/licenses/by/4.0/

Reviewer #1 (Remarks to the Author):

The authors report an interesting system for 4D printing using a DLP setup with a two-stage curing process: UV curing for acrylate and thiol groups, followed by thermal curing of epoxy moieties. They also demonstrate the applicability of their system through numerous examples. However, several modifications are necessary to support their claims. Below are my detailed comments:

Comment 1. *A more detailed analysis of the acrylate and thiol groups is necessary to substantiate the proposed two-stage curing mechanism.*

Response 1: Thanks for your great suggestion.

In the revised manuscript, we have conducted extensive Fourier Transform Infrared Spectroscopy (FTIR) characterizations to substantiate the proposed two-stage curing mechanism. Model reactions were carefully designed to investigate the possible reactions of acrylate, thiol, and epoxy groups during the three major stages of the DLP printing process, namely resin storage, UV polymerization during printing, and thermal treatment after printing. The temperature and UV irradiation conditions in these model reactions were kept identical to those used in the LCE printing process.

Because the new experimental results are substantial, we provide a brief summary of the main findings below to keep this response concise. All detailed FTIR data are presented and discussed in the revised Supplementary Materials (Section S3).

- During resin storage or during printing when the resin is not exposed to UV light, no unintended side reactions, such as epoxy–thiol, epoxy–amine, or Michael addition reactions, were detected for up to 21 hours of storage. The resin remains chemically stable and exhibits good shelf life.
- During UV polymerization, the reactions predominantly occur between the C=C bonds and thiol groups, which form a loosely crosslinked LCE network. The epoxy groups remain fully preserved for subsequent post-curing reactions.
- In the second-stage thermal polymerization, the epoxy–amine reaction becomes active, which generate additional covalent linkages that stabilize the programmed deformation of the printed LCE structures.

The new FTIR analyses are discussed in the revised manuscript on Page 5, as shown below:

In the Supplementary Material (Section S3), systematic Fourier Transform Infrared Spectroscopy (FTIR) analyses were conducted to substantiate the two-stage polymerization mechanism. Model reactions were carefully designed to examine the behavior of acrylate, thiol, and epoxy groups during resin storage, UV polymerization during DLP printing, and thermal treatment after printing. The results confirm that during resin storage, no unintended side reactions were detected for up to 21 hours. The printable ink remains chemically stable, likely due to the presence of toluene that suppresses unintended side reactions by diluting the reactive species. Upon UV irradiation, reactions predominantly occur between the C=C bonds and thiol groups, which form the crosslinked LCE network, while the epoxy groups remain fully preserved. In the second-stage thermal polymerization,

the epoxy–amine reaction becomes active, generating additional covalent linkages that stabilize the programmed deformation of the printed LCE structures.

Comment 2. *The structure during the first curing stage may correspond to a polydomain nematic state, which is not discussed in the manuscript. The actuated state at high temperature likely corresponds to the isotropic phase. These two states differ at the molecular level, and the recovered state at 100 °C may not replicate the first-stage state. The authors should provide an explanation of this, preferably with actual schematics—Figures 1d to 1h do not accurately represent the liquid crystalline state.*

Response 2: Thank you for catching this misrepresentation in the figure. You are correct that these two stages of the LCE exhibit the same macroscopic configuration but different molecular structures: one in the isotropic state, and the other in the polydomain state. In the revised manuscript, Figure 1d has been revised accordingly as below. The mesogens are now illustrated as locally aligned, with light gray lines added to represent their interactions in the polydomain state.

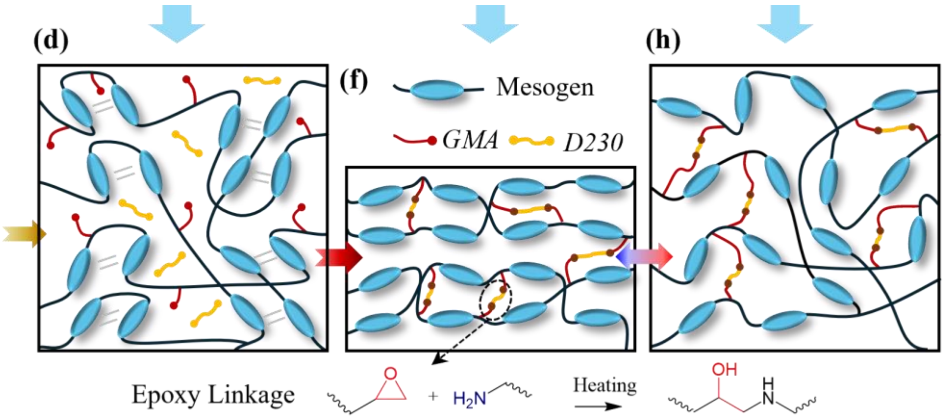

Furthermore, we have added the following sentence in the revised manuscript (Page 6) to highlight this difference.

Although the printed LCE recovers its as-printed macroscopic configuration upon heating, the two states differ in their molecular structures: the high-temperature state corresponds to the isotropic phase, whereas the LCE immediately after printing is in the polydomain state.

Comment 3. *Figure 2b does not show the actuation speed of the LCE specimens, despite the statement: "While Figure 2b compares the actuation speeds of LCE specimens over a fixed temperature range." Although actuation speed is indirectly represented in the cyclic test, this is not an appropriate way to discuss or quantify it.*

Response 3: Thank you for pointing this out. We agree with your comment. In the revised manuscript, this sentence has been removed to avoid any potential confusion. Please refer to our response to your Comment 4.

Comment 4. *The discussion in lines 198 to 226 is confusing and lacks clarity. The authors should revise this section to present their claims in a more structured and coherent manner.*

Response 4: Thank you for your comment. We agree that the discussions in these paragraphs were overly lengthy and lacked clarity. The main points we intended to convey are as follows:

(i) By examining the reversible actuation strain upon heating, the AE_6–AE_18 samples with moderate epoxy content were found to exhibit nearly identical onset shape recovery temperatures around 15 °C, whereas the onset temperatures for the AE_30 and AE_80 samples increased to 30–40 °C (Figure 2b).

(ii) The magnitude of actuation strain in the AE_6–AE_18 samples was close to the target programming strain of 80%, while the AE_30 and AE_80 samples exhibited much lower actuation strains due to excessive epoxy linkages restricting shape recovery (Figure 2d).

In the revised manuscript (on Page 9 and Page 10), the discussion has been condensed and focused on these main findings. Additionally, statements related to actuation speed have been removed to avoid potential confusion. The updated discussion in these paragraphs is provided below:

After programming, the evolution of actuation strain in AE_6–AE_80 samples was examined using DMA. Tests were conducted over a temperature range of –20 °C to 80 °C at a heating rate of 1 °C min^{-1}, as shown in ***Figure 2b****. Upon heating, the onset temperature of shape recovery varied with epoxy content. AE_6–AE_18 samples exhibited nearly identical onset temperatures around 15 °C, while AE_30 and AE_80 showed higher onset temperatures of approximately 30 °C and 40 °C, respectively. This indicates that epoxy contents above 30 % have a significant influence on the thermomechanical properties of LCEs.*

*During cooling, all specimens fully recovered their initial lengths, and this reversible shape-change behavior was highly repeatable over multiple heating–cooling cycles. For instance, AE_18 was subjected to 11 consecutive cycles and exhibited consistent strain evolution without noticeable degradation (****Figure 2c****).*

Figure 2d *summarizes the actuation strain amplitudes, which was obtained by heating the LCE samples at 80 ℃ for over 30 minutes until the strain reached saturation (Supplementary Materials, Section S7). The results show that the AE_1 specimen exhibited a much lower actuation strain due to its limited shape fixity, whereas AE_3–AE_18 samples achieved actuation strains close to the target programming strain. Increasing the epoxy content beyond 30 % led to a pronounced reduction in actuation strain; for example, AE_80 showed a strain amplitude of only ~18 %. This sample is not able to fully recover its initial printed geometry because the newly formed epoxy linkages during programming are stress-free; at high concentrations, these linkages strongly resist the entropy-driven recovery of the deformed acrylate network.*

Comment 5. *Some figures are explained out of order, which disrupts the logical flow of the manuscript.*

Response 5: Thank you for pointing this out. We have carefully revised the manuscript and made the necessary corrections. All figures are now explained in the proper order throughout the text.

Reviewer #2 (Remarks to the Author):

Comment 1. *This manuscript presents many interesting results and images. However, from a chemical standpoint, as shown in Figure 1(b), the monomer mixture consists of diacrylate, dithiol, diamine, and epoxy monomers. In the first stage, the dithiol and diacrylate undergo free-radical polymerization, while the epoxy groups are expected to react simultaneously in the presence of diamine. Therefore, the reaction pathway may not match the one described in Figures 1(d) and (f). The authors should provide FTIR evidence to demonstrate the evolution of functional groups throughout the entire programming process. Furthermore, since epoxy groups (GMA) can undergo ring-opening reactions under free-radical conditions, the authors must explain how the epoxy rings are preserved during the initial polymerization stage. Meanwhile, the acrylate–thiol Michael addition proceeds in the presence of basic amines.*

Response 1: We sincerely appreciate the reviewer's insightful comment on the chemical reaction mechanisms, which has greatly helped improve the clarity and quality of our study. In the revised manuscript, we have performed extensive FTIR characterizations to substantiate the proposed two-stage curing mechanism. Model reactions were carefully designed to investigate the possible reactions of acrylate, thiol, and epoxy groups during the three major stages of the DLP printing process, namely resin storage, UV polymerization during printing, and thermal treatment after printing. The temperature and UV irradiation conditions in these model reactions were kept identical to those used in the LCE printing process.

Because the new experimental results are substantial, we provide a brief summary of the main findings below to keep this response concise. All detailed FTIR data are presented and discussed in the revised Supplementary Materials (Section S3).

The key findings and our response to your comments are summarized below:

- **Under ambient conditions at room temperature**, we confirmed that the epoxide groups in GMA did not react with the amine groups in D230 during 21 hours of storage, nor did they react with the thiol groups under the catalytic influence of amines. This conclusion was drawn from a series of model reactions. Furthermore, the printable resin containing all components, including catalysts, photoinitiator, and toluene solvent, was examined after storage at room temperature for 21 hours. In a separate test, the mixture was heated to 80 °C for 30 minutes. No observable changes were detected in any characteristic FTIR peaks in either case, particularly those corresponding to the C=C bonds, thiol groups, and epoxy rings. These results confirm that the acrylate–thiol Michael addition does not occur in the presence of amines and that the printable resin remains chemically stable at room temperature.

  Although the reactions mentioned by the reviewer are thermodynamically favorable, their reaction kinetics is very slow during the room-temperature resin storage or during DLP printing when the resin is not exposed to UV light. The reaction rate becomes much faster only upon heating, such as at 80 °C as used in this study. Furthermore, this stability is attributed to the presence of toluene solvent in the printable ink. In this work, a moderate

amount of toluene (30 wt%) was added to the ink to reduce its viscosity for DLP printing while avoiding noticeable volume shrinkage of the printed LCE after thermal treatment. The toluene solvent suppresses these unintended side reactions by diluting the reactive species and increasing the intermolecular distances between them, thereby enhancing the shelf life of the printable ink.

- **During the UV polymerization step in DLP printing**, FTIR analyses of the model reactions revealed that the reactions predominantly occur between the C=C bonds and thiol groups through radical homo-polymerization of the double bonds and thiol–ene reactions, which form a crosslinked LCE network. The epoxy groups did not react with the thiol groups under free-radical conditions; instead, they remained chemically inert and were fully preserved for subsequent post-curing reactions.

  These results also indicate that, under the UV irradiation conditions used in DLP printing, the homo-polymerization of C=C bonds exhibits higher reactivity than the thiol–ene reaction with thiol groups. This finding justifies the use of an excess amount of C=C bonds in the resin formulation relative to thiol groups (see our response to the next comment), which prevents excessive unreacted thiol functionalities after printing.
- **During the second-stage thermal polymerization**, we confirmed that the epoxy–amine reaction become active, which forms additional covalent linkages that stabilize the programmed deformation of the printed LCE structures.

The new FTIR analyses are discussed in the revised manuscript on Page 5, as shown below:

> *In the Supplementary Material (Section S3), systematic Fourier Transform Infrared Spectroscopy (FTIR) analyses were conducted to substantiate the two-stage polymerization mechanism. Model reactions were carefully designed to examine the behavior of acrylate, thiol, and epoxy groups during resin storage, UV polymerization during DLP printing, and thermal treatment after printing. The results confirm that during resin storage, no unintended side reactions were detected for up to 21 hours. The printable ink remains chemically stable, likely due to the presence of toluene that suppresses unintended side reactions by diluting the reactive species. Upon UV irradiation, reactions predominantly occur between the C=C bonds and thiol groups, which form the crosslinked LCE network, while the epoxy groups remain fully preserved. In the second-stage thermal polymerization, the epoxy–amine reaction becomes active, generating additional covalent linkages that stabilize the programmed deformation of the printed LCE structures.*

Comment 2*. As shown in Table S1, the overall molar ratio between the acrylate and epoxy networks varies across the samples, ranging from 1:0 to 1:0.8. Under these conditions, the excess diacrylate would also undergo free-radical polymerization during the first reaction stage. Given this, what is the rationale for incorporating an excess amount of diacrylate? If the programmed polymerization pathway differs from that illustrated in Figures 1(d) and (f), the validity of the results presented in this manuscript becomes questionable.*

Response 2: We appreciate the reviewer's thoughtful comment. The purpose of introducing an excessive amount of diacrylate is to tune the thermomechanical properties and shape-changing performance of the material.

As evidenced by our FTIR results, during the first-stage UV polymerization, the diacrylate groups are expected to be fully reacted, whereas the epoxy rings are largely preserved for the second-stage polymerization. Therefore, the molar ratio between the acrylate and epoxy components can be intentionally adjusted to tailor the material properties. As shown in Figure 2, to obtain two-stage LCEs that can both fix the programmed shape and remain sufficiently soft to fully recover their original shape upon heating, the epoxy content must be relatively low, ideally within 6–12 mol% relative to the acrylate content. If the epoxy content equals that of the diacrylate (i.e., 100% epoxy), the resulting material behaves as a very stiff plastic without the LCE characteristics of programmability and reversible actuation performance.

On the other hand, if the reviewer's reference to "an excess amount of diacrylate" is in comparison to the thiol groups in each LCE sample (see Table S1), this choice is also well justified. As detailed in the FTIR results for the UV polymerization stage, the C=C bonds exhibit higher reactivity than the thiol groups. If identical molar amounts of acrylate and thiol groups are used in the resin mixture, a substantial number of thiol groups are likely to remain unreacted after UV polymerization. These residual thiol groups are not required for the second-stage polymerization to fix the programmed shape of the LCEs; instead, they may complicate the system and compromise the mechanical integrity of the material. Therefore, an excess amount of diacrylate was intentionally included to minimize the presence of unreacted thiol groups.

To clarify this point for readers, we have added the following sentences in the revised manuscript (Page 7):

> *"Across all LCE samples, there is an excess of acrylate relative to the thiol groups. As shown in the FTIR characterizations in the Supplementary Materials (Section S3), during UV polymerization, the acrylate bonds exhibit higher reactivity than the thiol groups. An excess amount of acrylate helps minimize residual thiol groups, which are not required for the second-stage thermal polymerization"*

Together with the FTIR characterizations presented in our Response 1, these results support that the polymerization pathway follows that illustrated in Figures 1(d) and 1(f), which confirm that the conclusions of this study are valid.

Comment 3. *The proposed two-stage polymerization is designed based on the stoichiometry of functional groups. However, from a chemical standpoint, the feasibility of the designed functional group reactions is questionable. As this issue is central to the manuscript, it must be thoroughly clarified before the manuscript can be considered for further review.*

Response 3: We sincerely appreciate the reviewer's comment regarding the feasibility of the proposed two-stage polymerization mechanism. Please kindly refer to our detailed response to Comment 1 and the FTIR results in the revised Supplementary Materials (Section S3). These

characterizations demonstrate that the potential side reactions raised by the reviewer did not occur under the conditions of our study. Therefore, it remains valid to design the polymer network based on the stoichiometric ratios of the functional groups involved. We hope that these additional results and clarifications have fully addressed the reviewer's concern.

Comment 4. *As described in the experimental section, RM257, GMA, EDDET, and D230 were mixed and stored at room temperature in the dark for 12 hours. Theoretically, in the presence of amines, acrylate–thiol Michael addition should proceed even at room temperature. How, then, is the occurrence of this reaction controlled?*

Response 4: We agree with the reviewer that the acrylate–thiol Michael addition is thermodynamically favorable; however, its occurrence strongly depends on the presence of sufficiently basic catalysts, such as tertiary amidines or phosphines. In contrast, the primary amine groups in D230 possess much lower basicity and nucleophilicity, making their catalytic effect on the thiol–(meth)acrylate Michael addition negligible under the conditions of this study.

As demonstrated in our FTIR analyses (see Response 1 and the revised Supplementary Materials, Section S3), this reaction did not occur under the conditions of our study. First, in the model reaction containing GMA, EDDET, and D230 at room temperature, the absorption peak corresponding to the thiol groups remained unchanged after 6 hours of storage (Figure S2). Second, we monitored the FTIR spectra of the complete LCE resin formulation containing all components for up to 21 hours of storage at room temperature, as well as after heating at 80 °C for 30 minutes. No notable changes were observed in any of the characteristic absorption peaks (Figure S3), which confirm that this side reaction was effectively suppressed.

Another important factor contributing to this stability is the presence of toluene solvent in the printable ink. As mentioned earlier in our response, toluene was introduced to reduce the viscosity for DLP printing. It simultaneously dilutes the reactive species and increasing their intermolecular distances, thereby inhibiting undesired reactions.

Comment *5. This manuscript presents many interesting results and images based on the proposed two-stage polymerization. However, from a theoretical standpoint, the feasibility of the controlled two-stage polymerization for the designed monomer mixtures is questionable. Spectral evidence supporting the proposed reactions at each stage must be provided. How are the functional groups controlled during the process? How is the epoxy ring prevented from opening in the presence of basic amines or under free-radical polymerization conditions? What mechanisms ensure that epoxy ring opening does not occur during free-radical reactions? Furthermore, why does excess acrylate not undergo polymerization during the free-radical stage?*

Response 5: We hope that the spectral evidence provided for each stage has fully addressed the reviewer's concerns. These results offer direct and comprehensive proof that each functional group behaves in a controlled and predictable manner throughout the two-stage polymerization. Our responses to the individual points in this comment are provided below.

(1) Control of functional groups during the process

The FTIR analyses confirm that each functional group reacts selectively during its designated stage. During resin storage or during DLP printing when the resin is not exposed to UV light, the mixture remains chemically stable for at least 21 hours. In the first-stage UV polymerization, only the acrylate and thiol groups participate in the photopolymerization, while the epoxy and amine groups remain inactive. In the second-stage thermal polymerization, the epoxy and amine groups react to form additional covalent linkages that fix the programmed deformation.

(2) Mechanisms preventing epoxy ring opening under free-radical and amine conditions

The prevention of epoxy ring opening under these conditions is primarily governed by kinetic and energetic factors. During resin storage, although both epoxy and amine groups are present, the activation energy for their reaction is relatively high. In addition, the presence of toluene solvent dilutes the reactive species, which further decreases the reaction rate. As a result, the reaction does not proceed appreciably unless the system is heated. The FTIR spectra show no notable changes in the epoxy peak over a 21-hour period, which indicates that the reaction does not occur at room temperature.

During the UV polymerization step in DLP printing, each printed layer is exposed to UV light for approximately 15 seconds. Within this short exposure time, the acrylate and thiol groups react much more rapidly through free-radical mechanisms, which consume the available radicals before any epoxy ring-opening reactions can occur. Therefore, both the kinetic limitations and the competition for reactive species effectively prevent epoxy ring opening during this stage.

(3) Polymerization of excess acrylate during the free-radical stage

Indeed, the excess acrylate groups undergo polymerization during the free-radical stage. FTIR characterizations indicate that nearly all acrylate double bonds are consumed during the first-stage UV polymerization. The second-stage polymerization involves only the epoxy–amine reaction. Excess acrylate groups are intentionally introduced into the system to tune the thermomechanical properties of the material (please see our Response 2 for details).

Comment 6. *During the thermal treatment, the authors mentioned that LCE materials tended to align along the local stress directions, resulting in a monodomain in the LCE. It is recommended to explain the phenomenon in a more detailed way with the support of schematic illustrations or further experimental results.*

Response 6: We believe that this concern arises from a misunderstanding caused by the wording in our original manuscript. During the thermal treatment, it is not the heating process itself that aligns the mesogens, but rather the global mechanical deformation applied during the programming step.

When a macroscopic programming deformation is applied to the LCE structure, the internal mesogens at each point tend to align along the local principal stress direction, as demonstrated in our previous experimental study (Ref. 37 in the manuscript). Because different regions of the structure experience different principal stress states, the mesogen alignment directions vary

spatially. Thus, the LCE does not form a uniform monodomain but instead exhibits locally aligned regions whose orientations follow the distribution of principal stresses within the material. In this process, the role of heating is to activate the epoxy–amine reaction, which covalently fixes the programmed deformation.

To clarify this point to the readers, the following sentences have been updated in the revised manuscript on Page 4.

In the programming stage, the printed lattice structure was compressed to 50% global engineering strain, which drove the internal mesogens at each point of the printed LCE structures to align along the local principal stress direction [37]. Because different regions of the structure experience different principal stress states, the mesogen alignment directions varied spatially.

Reviewer #3 (Remarks to the Author):

The manuscript presents a 4D printing method for liquid crystal elastomers (LCEs) using vat photopolymerization via a two-stage curing process. This newly developed approach enables high-resolution 4D printing of LCEs, representing a significant advancement for engineering applications. The manuscript is well organized. However, several questions remain to be addressed.

***Comment** 1. Compared to direct ink writing (DIW), the DLP method offers a significant advantage in resolution. It is suggested to include the printing resolution data and add the comparison in the manuscript.*

Response 1: Thank you for this valuable suggestion.

In the revised manuscript, we investigated the printing resolution of our DLP setup. Specifically, we use the DLP to print several cylinders with diameters ranging from 1.4 mm to 0.05 mm. The corresponding CAD models and printed results are shown in the following figure. It is observed that the smallest feature that can be accurately fabricated is approximately 0.3 mm in diameter; cylinders below this size either cannot be printed or exhibited poor surface quality. This critical feature size is notably smaller than that achievable by DIW printing, where the printable resolution is primarily limited by the nozzle diameter, typically on the millimeter scale.

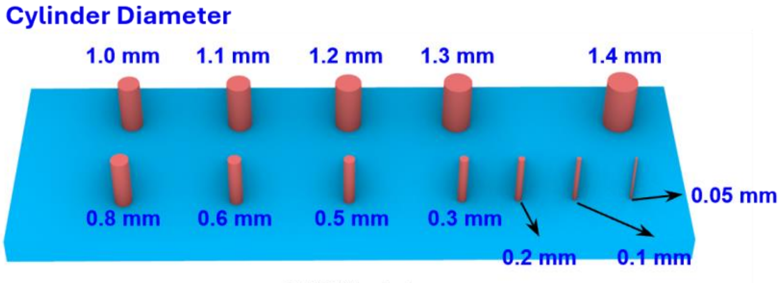

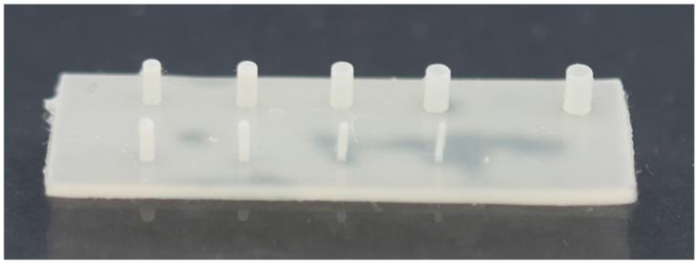

Printed Structure

It should also be noted that the printing resolution of DLP systems depends strongly on the printer hardware, particularly the light engine. In this work, we used a commercially available 405 nm optical engine (Wintech, Carlsbad, CA), which provides a representative resolution for standard DLP setups. Higher resolutions could be achieved by employing optical engines with finer projection pixel sizes.

The characterization of the DLP printing resolution has been added to the revised Supplementary Materials (Section S1). The following sentences are also added to the revised

manuscript on Page 4:

The adopted DLP printer not only enables the fabrication of intricate LCE components without the need for support materials but also offers a much higher printing resolution compared to the conventional DIW method. For example, as shown in the Supplementary Material (Section S1), the smallest printable feature size is approximately 0.3 mm.

Comment 2. *Two-stage polymerization process represents the most widely used strategy for 4D printing of LCEs. The critical aspect lies in the distinct reaction rates or reaction conditions between the two polymerization stages. Otherwise, it becomes challenging to achieve the reproducible programming of LCEs. It is known that thiol-epoxy reaction can also happen in the ambient condition. How about the reaction kinetics of the thiol-acrylate? Since the 3D printing time for the objects in different sizes is different significantly. It is important to study the reaction kinetics of thiol-epoxy reaction.*

Response 2: Thank you very much for your thoughtful comments regarding the chemical reactions. In the revised manuscript, we have conducted extensive FTIR characterizations to investigate the reaction pathways occurring during the three major stages of the DLP printing process: resin storage, UV polymerization during DLP printing, and subsequent thermal treatment. These analyses involved monitoring the evolution of FTIR absorption peaks through a series of model reactions designed to replicate the actual processing conditions used in DLP printing. All detailed FTIR data are presented and discussed in the revised Supplementary Materials (Section S3). Our major findings are summarized below:

I. During resin storage under ambient conditions or during DLP printing when the resin is not exposed to UV light, the epoxy rings did not noticeably react with the thiol groups in the presence of amine catalysts. This was confirmed using a model system containing GMA (epoxy groups), D230 (amine groups), and EDDET (thiol groups) mixed at the same molar ratio as in the printable ink of the AE_80 sample. The FTIR absorption peaks were monitored for 6 hours at room temperature. As shown in Figure SX, the epoxy peak exhibited only a slight decrease, within the range of experimental variation, while the thiol peak remained unchanged. This indicates that the epoxy–thiol reaction is negligible under these conditions. Importantly, the actual DLP printing process in this study takes less than one hour, which is significantly shorter than the 6-hour FTIR observation period.

II. The printable ink containing all components, including catalysts, photo-initiator, and toluene solvent, was also examined after stabilization at room temperature for 21 hours. In a separate test, the mixture was heated to 80 °C for 30 minutes. No observable changes were detected in any characteristic FTIR peaks in either case, particularly those corresponding to C=C bonds, thiol groups, and epoxy rings. These results confirm that the printable resin remains chemically stable and that thiol–epoxy and thiol–acrylate reactions do not occur under ambient conditions.

III. To further confirm that the epoxy groups do not react with thiol groups under free-radical

conditions during UV exposure, another model reaction was performed by mixing GMA, EDDET, and the photo-initiator TPO. This mixture simultaneously contained C=C, thiol, and epoxy functional groups. The corresponding FTIR spectra (Figure SX) show that the C=C bonds were completely consumed and the thiol peak decreased significantly after UV irradiation. However, the epoxy peak at 907 cm^{-1} remained unchanged. These results confirm that during the UV polymerization step in DLP printing, the thiol–acrylate reaction dominates the network formation due to its much faster kinetics, while the epoxy groups remain chemically inert and are preserved for the subsequent second-stage thermal polymerization.

In conclusion, the printable resin is chemically stable during storage under ambient conditions, with no noticeable thiol–epoxy or thiol–acrylate reactions. This stability is attributed to the presence of toluene solvent in the printable ink. In this work, a moderate amount of toluene (30 wt%) was added to the ink to reduce its viscosity for DLP printing while avoiding noticeable volume shrinkage of the printed LCE after thermal treatment. The toluene solvent suppresses unintended side reactions by diluting the reactive species and increasing the intermolecular distances between them, thereby enhancing the shelf life of the printable ink. During UV polymerization, the thiol–acrylate reaction proceeds much faster than the thiol–epoxy reaction. Within this short UV exposure time (~15 seconds per layer), the acrylate and thiol groups rapidly react through free-radical mechanisms, consuming the available radicals before any epoxy ring-opening reactions can occur. Consequently, the epoxy rings remain fully preserved for the second-stage polymerization.

The new FTIR analyses are discussed in the revised manuscript on Page 5, as shown below:

In the Supplementary Material (Section S3), systematic Fourier Transform Infrared Spectroscopy (FTIR) analyses were conducted to substantiate the two-stage polymerization mechanism. Model reactions were carefully designed to examine the behavior of acrylate, thiol, and epoxy groups during resin storage, UV polymerization during DLP printing, and thermal treatment after printing. The results confirm that during resin storage, no unintended side reactions were detected for up to 21 hours. The printable ink remains chemically stable, likely due to the presence of toluene that suppresses unintended side reactions by diluting the reactive species. Upon UV irradiation, reactions predominantly occur between the C=C bonds and thiol groups, which form the crosslinked LCE network, while the epoxy groups remain fully preserved. In the second-stage thermal polymerization, the epoxy–amine reaction becomes active, generating additional covalent linkages that stabilize the programmed deformation of the printed LCE structures.

***Comment 3**. The curing temperature for the amine-epoxy is higher than the phase transition temperature of 3D printed elastomer. At this higher temperature, the LCEs before thermal curing is in the isotropic state. Will this affect the aligning process?*

Response 3: We thank the reviewer for this excellent question.

We believe that the effect of heating on the mesogen alignment process is negligible. During the programming stage, the global deformation is fixed, so the microscale network structure and chain configurations remain stable. Although heating above the phase transition temperature of LCEs reduces the intermolecular interactions among the mesogens, their orientations and positions remain largely unchanged because the chain configurations and network structure are constrained by the imposed displacement boundary conditions. As a result, the overall spatial mesogen alignment is maintained during the thermal curing process.

To clarify this point to the readers, the following statement is added to the revised manuscript on Page 5:

> *"It is noted that during the programming stage, the global deformation is fixed, which stabilizes the microscale network structure and chain configurations. Although heating above the phase transition temperature reduces the intermolecular interactions among the mesogens, their orientations and positions remain largely unchanged because the chain configurations and network structure are constrained by the imposed displacement boundary conditions. As a result, the overall spatial mesogen alignment is maintained during the thermal curing process."*

Comment 4*. On page 6, the authors said that "A relatively low processing temperature of 80 °C was selected to minimize the risk of thermal degradation". It may be convincible that the TGA results are provided. Common polymeric materials typically undergo thermal degradation above 300 C. Compared to the degradation temperature, the phase transition temperature of the firstly crosslinked LCEs represents a more critical parameter for the second curing process.*

Response 4:

Following the reviewer's suggestion, we performed Thermogravimetric Analysis (TGA) on the LCE sample AE_6. The change in normalized mass with temperature is shown below. As the reviewer anticipated, the thermal degradation temperature, defined as the temperature corresponding to a 5% mass loss, is approximately 330 °C, which is significantly higher than the programming temperature used in this study. This new data is added to the revised Supplementary Materials (Section S2)) and referred in the main text on Page 5.

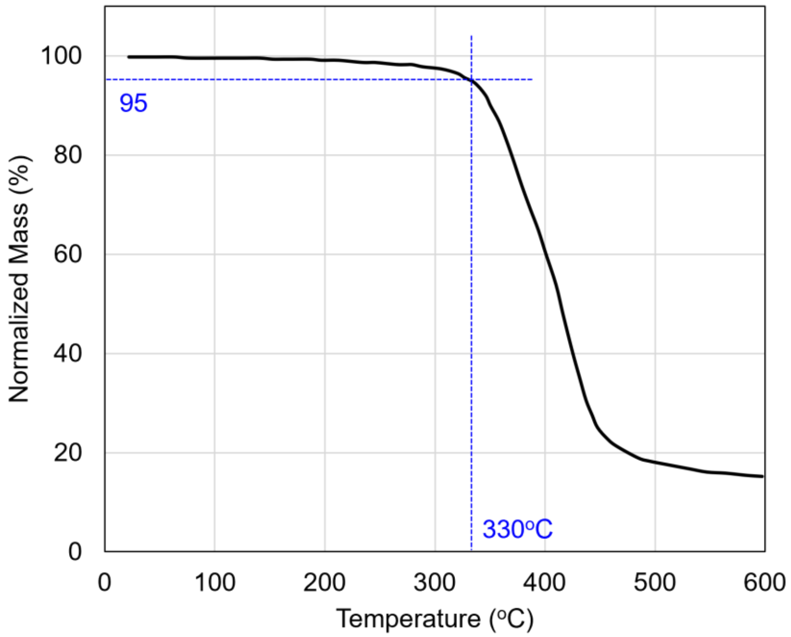

Regarding the selection of the programming temperature, we agree that the phase transition temperature of the LCE represents a more critical factor to consider. The programming temperature must be at least above this transition temperature to provide sufficient chain mobility for the epoxy–amine reactions to proceed effectively.

To clarify this point for readers, the following statement has been added to the revised manuscript (Page 5):

The programming temperature must be carefully selected. On one hand, the programming temperature should be higher than the phase transition temperature of the LCE to ensure sufficient chain mobility for the epoxy–amine reaction. On the other hand, increasing the temperature accelerates the reaction rate according to the Arrhenius law, thereby improving the programming efficiency. However, excessively high temperatures may lead to thermal degradation of the elastomeric LCEs. As revealed by Thermogravimetric Analysis (TGA) tests provided in the Supplementary Materials (Section S2), the onset of degradation occurs at approximately 330 °C. In this study, a relatively low processing temperature of 80 °C was selected to avoid any risk of thermal degradation, while a prolonged heating duration of 14 hours was used to ensure complete curing of epoxy.

Comment *5. Compression programming is employed to configure the actuation behavior of 3D-printed LCE metamaterials. However, the initially crosslinked LCE network typically exhibits low mechanical stiffness, raising concerns about structural instability during compression. How to solve this problem?*

Response 5: Thank you for this insightful comment. It raises an important practical consideration.

When compressing the metamaterials, the structure may experience a risk of global buckling,

particularly when the transverse dimension is much smaller than the height of the structure. To address this issue, one possible solution is to apply transverse stretching instead of compression to achieve an equivalent global programming deformation. If this approach results in deviations or is not feasible, a more standard method is to employ an anti-buckling compression fixture, as recommended in ASTM D695. In such a setup, the specimen is enclosed by a rigid frame providing continuous lateral support, while its motion is guided by low-friction rails. This configuration ensures uniform transverse displacement during large-strain compression and effectively suppresses localized bulging or buckling.

We believe this is an important point for readers' understanding. The following statement has been added to the revised manuscript on Page 14:

"During the compression programming of the auxetic metamaterials, no noticeable structural instability was observed in this study. However, for structures whose transverse dimension is much smaller than their height, global buckling may occur. To address this issue, one possible approach is to apply transverse stretching instead of compression to achieve an equivalent global programming deformation. Alternatively, a standardized method is to use an anti-buckling compression fixture (e.g., ASTM D695), which employs a rigid frame to provide continuous lateral support and ensure uniform transverse displacement while preventing localized bulging or buckling."

Comment 6. *How about the parameters used in the FEA simulations? The mechanical parameters should be measured in appropriate temperatures.*

Response 6: In this study, the FEA simulations were performed using the AE_6 sample. As described in the Supplementary Material (Section S10), the material was modeled as a linear elastic solid. For simulations conducted at room temperature, such as those examining the auxetic metamaterials, a constant elastic modulus of 1.05 MPa and a Poisson's ratio of 0.49 were used. The modulus was obtained from uniaxial tensile tests of the AE_6 sample after thermal treatment, as presented in Figure 1l of the main text.

We acknowledge that the linear elastic assumption does not capture the nonlinear stress–strain behaviors of LCEs. Nevertheless, the simulated results in Figure 3f show close agreement with the experimental results. This consistency is likely because the global compression strain of the metamaterial is only 24%, and most finite elements experience relatively small deformations. Only a limited number of elements near the holes develop localized stress concentrations, which have a minor influence on the overall deformation pattern. Therefore, the simplified linear elastic assumption does not significantly affect the accuracy of the predicted global shape of the metamaterial.

For simulations involving temperature variations, such as the folding actuation shown in Figure 2i and the temperature-dependent stiffness of the LCE lattice shown in Figure 3b, the modulus values at different temperatures were derived from DMA data. As shown in Figure S10d, the modulus of the AE_6 sample after thermal treatment decreases slightly from ~3 MPa at room

temperature to ~1 MPa at 80 °C. It should be noted that the modulus measured by DMA at room temperature (3 MPa) differs slightly from that obtained by uniaxial tension (1.05 MPa), which is expected due to differences in the testing methods.

To maintain consistency across all FEA simulations, the room-temperature modulus obtained from the uniaxial tensile test (1.05 MPa) was used as the reference value. The temperature-dependent modulus from DMA was then applied to scale this reference modulus. Specifically, the modulus used in the FEA at each temperature was obtained by multiplying the tensile test modulus by the ratio of the DMA-measured modulus at that temperature to the DMA-measured modulus at room temperature. The resulting temperature-dependent modulus data were input into ABAQUS as a temperature-dependent property. This approach ensures that the simulations consistently reflect the experimentally observed stiffness variation with temperature while maintaining a unified reference modulus.

To clarify this point to the readers, the following statement is added to the revised Supplementary Material, Section S10, when we describe the FEA details.

> *In this study, the FEA simulations were performed using the AE_6 sample. The material was modeled as a linear elastic solid. For simulations conducted at room temperature, such as those examining the auxetic metamaterials, a constant elastic modulus of 1.05 MPa and a Poisson's ratio of 0.49 were used. The modulus was obtained from uniaxial tensile tests of the AE_6 sample after thermal treatment, as presented in Figure 1l of the main text.*
>
> *For simulations involving temperature variations, the modulus values at different temperatures were derived from DMA data. As shown in Figure S10d, the modulus of the AE_6 sample after thermal treatment decreases slightly from ~3 MPa at room temperature to ~1 MPa at 80 °C. It should be noted that the modulus measured by DMA at room temperature differs slightly from that obtained by uniaxial tension, which is expected due to differences in the testing methods. To maintain consistency across all FEA simulations, the room temperature modulus obtained from the uniaxial tensile test (1.05 MPa) was used as the reference value. The temperature-dependent modulus from DMA was then applied to scale this reference modulus. Specifically, the modulus used in the FEA model at each temperature was obtained by multiplying the tensile test modulus by the ratio of the DMA-measured modulus at that temperature to the DMA-measured modulus at room temperature. The resulting temperature-dependent modulus data were input into ABAQUS as a temperature-dependent property. This approach ensures that the simulations consistently reflect the experimentally observed stiffness variation with temperature while maintaining a unified reference modulus.*

***Comment** 7. Polyphenylene oxide (PPO) is conventionally known as a high-performance engineering polymer, not a photoinitiator. Please check it.*

Response7: We thank the reviewer for catching this typo. The photo-initiator used in this study is diphenyl(2,4,6-trimethylbenzoyl)phosphine oxide (TPO), not PPO. This has been corrected in the revised manuscript.

***Comment 8**. On Page 5, the authors said that "This complete shape fixity was attributed to the ring-opening polymerization between GMA and the unreacted D230". The "ring-opening polymerization" usually refers to a type of chain-growth polymerization where cyclic monomers are attacked by the terminus of a polymer chain, leading to the formation of longer polymer chains. The amine-epoxy reaction here is a ring-opening reaction instead of ring-opening polymerization.*

Response 8: We thank the reviewer for this correction. In the revised manuscript, all instances of "ring-opening polymerization" have been replaced with "ring-opening reaction" to ensure accuracy.

Reviewer #3 (Remarks to the Author):

The authors have thoroughly addressed the major concerns. The manuscript is significantly improved. I recommend acceptance of the manuscript after minor revision. Minor suggestions:

Comment 1: *It is very interesting that the printable ink, which contains highly reactive components (amine catalysts, thiol, and epoxy groups), remains stable even after being heated to 80 °C for 30 minutes. This exceptional stability is highly counterintuitive considering the known rapid reaction kinetics of thiol-epoxy systems in the presence of amine catalysts. To validate this claim, I strongly recommend the authors provide additional supporting evidence, for instance, performing 1H NMR spectroscopy on the ink composition before and after treatment.*

Response 1: I apologize for the confusion caused by my previous writing. It was my oversight that this specific point was not communicated clearly between my student researchers and me, and it was therefore not clearly articulated in the Supplementary Materials.

The FTIR spectrum shown in Figure S5c was not obtained from the final printable ink containing all reactive components. Instead, it was measured from a precursor resin that only contains RM257, EDDET, and the photoinitiator TPO. This precursor mixture corresponds to an intermediate step in the ink preparation process.

During ink preparation, RM257, EDDET, TPO, and toluene are first mixed at their designated ratios in a glass vial and heated at 80 °C for 30 minutes to fully dissolve the solid powders. After the solution is cooled back to room temperature, the epoxy-containing components (GMA and D230) are added to obtain the final printable ink. The FTIR test in Figure S5c was therefore performed only on the precursor mixture, prior to adding the epoxy components. The purpose was to confirm that no notable chemical reactions occur during the 80 °C heating step. The nearly identical FTIR spectra before and after heating indicate that this precursor resin remains chemically stable under these conditions. In contrast, if all components (including the epoxy and amine) are mixed and then heated at 80 °C, as you expected, substantial chemical reactions will occur because of the high reactivity among the amine, epoxy, and thiol groups.

Please be noted that FTIR tests in Figure S5a and Figure S5b are still for the final printable ink containing all reactive components.

In the revised Supplementary Materials (Section S3), the discussion related to Figure S5c has been carefully revised as follows to make clear that the FTIR data were collected from the precursor mixture rather than from the complete printable ink.

> *"In a separate test, a precursor resin was prepared by mixing RM257, EDDET, and TPO at the same ratios as used in the printable ink. FTIR absorption spectra were recorded before and after heating at 80 °C for 30 minutes. This precursor mixture corresponds to an intermediate step in the ink preparation process, before mixing with epoxy content for DLP printing (see the Methods section of the main text). As shown in* ***Figure S5c****, the FTIR spectrum remains*

essentially unchanged, indicating that no notable chemical reactions occur during the 80 °C heating step. It is noted that, in contrast, if all components are mixed and then heated at 80 °C, substantial chemical reactions are expected because of the high reactivity between the amine, epoxy, and thiol."

We also refined the Experimental Section in the main manuscript to correct any inaccurate descriptions. The revised description is:

"To prepare the printable ink, RM257, EDDET, TPO, and toluene were first mixed at their designated ratios in a glass vial and heated at 80 °C for 30 minutes to fully dissolve the solids. After removal from the oven, the mixture was allowed to cool to room temperature. GMA and D230 were then added in their respective ratios and thoroughly mixed …"

Comment 2: *Regarding the authors' response to Comment 4 from Reviewer 3, a clarification on the programming temperature is warranted. It is not strictly necessary for the programming temperature to exceed the phase transition temperature. The chain mobility is sufficient enough when temperature is higher than the glassy transition temperature.*

Response 2: Sorry I misinterpreted your previous comment.

In this study, the programming temperature is defined as the temperature that not only allows deformation of the material but also activates the epoxy–amine reaction so that the programmed shape can be fixed. We agree that, if we just want to deform the material, even room temperature would be sufficient, as long as the temperature exceeds the glass transition temperature and provides adequate chain mobility. However, to accelerate the chemical reaction, the sample needs to be heated. Based on our tests, heating at 80 °C for 14 hours provides an effective balance between reaction rate and material stability, and these conditions were therefore adopted in our experiments.

The previous statement that we added to the manuscript has been further revised as follows to make this clearer to the readers.

"The programming temperature must be carefully selected, which should not only allow deformation of the material but also activate the epoxy–amine reaction so that the programmed shape is fixed. An increase in programming temperature accelerates the reaction rate according to the Arrhenius law and thus improves programming efficiency. However, excessively high temperatures may cause thermal degradation of the elastomeric LCEs. Thermogravimetric analysis (TGA) in the Supplementary Materials (Section S2) shows that degradation starts at approximately 330 °C. In this study, a relatively low processing temperature of 80 °C was selected to avoid the risk of thermal degradation while still providing sufficient reaction kinetics for effective programming."
